# Neurocognitive outcomes in Malawian children exposed to malaria during pregnancy: An observational birth cohort study

**DOI:** 10.1371/journal.pmed.1003701

**Published:** 2021-09-28

**Authors:** Andrea M. Weckman, Andrea L. Conroy, Mwayiwawo Madanitsa, Bruno Gnaneswaran, Chloe R. McDonald, Linda Kalilani-Phiri, Jaya Chandna, Doreen Ali, Victor Mwapasa, Carole Khairallah, Kyaw Lay Thwai, Steven R. Meshnick, Steve M. Taylor, Feiko O. ter Kuile, Kevin C. Kain, Melissa Gladstone

**Affiliations:** 1 Department of Laboratory Medicine and Pathobiology, University of Toronto, Toronto, Ontario, Canada; 2 SAR Laboratories, Sandra Rotman Centre for Global Health, Toronto General Hospital Research Institute, University Health Network, Toronto, Ontario, Canada; 3 Department of Pediatrics, Indiana University School of Medicine, Indianapolis, Indiana, United States of America; 4 College of Medicine, University of Malawi, Blantyre, Malawi; 5 Academy of Medical Sciences, Malawi University of Science and Technology, Thyolo, Malawi; 6 Women and Children’s Health, Institute of Life Course and Medical Sciences, University of Liverpool, Liverpool, United Kingdom; 7 Grand Challenges Canada, Toronto General Hospital, University Health Network, Toronto, Ontario, Canada; 8 Department of Infectious Disease Epidemiology, London School of Hygiene & Tropical Medicine, London, United Kingdom; 9 Department of Preventive Health Services, Ministry of Health, Lilongwe, Malawi; 10 Department of Clinical Sciences, Liverpool School of Tropical Medicine, Liverpool, United Kingdom; 11 Department of Epidemiology, Gillings School of Global Public Health, University of North Carolina at Chapel Hill, Chapel Hill, North Carolina, United States of America; 12 Division of Infectious Diseases, Duke University, Durham, North Carolina, United States of America; 13 Duke Global Health Institute, Duke University, Durham, North Carolina, United States of America; 14 Tropical Disease Unit, Division of Infectious Diseases, Department of Medicine, University of Toronto, Toronto, Ontario, Canada; Instituto de Salud Global de Barcelona, SPAIN

## Abstract

**Background:**

Annually 125 million pregnancies are at risk of malaria infection. However, the impact of exposure to malaria in pregnancy on neurodevelopment in children is not well understood. We hypothesized that malaria in pregnancy and associated maternal immune activation result in neurodevelopmental delay in exposed offspring.

**Methods and findings:**

Between April 2014 and April 2015, we followed 421 Malawian mother–baby dyads (median [IQR] maternal age: 21 [19, 28] years) who were previously enrolled (median [IQR] gestational age at enrollment: 19.7 [17.9, 22.1] weeks) in a randomized controlled malaria prevention trial with 5 or 6 scheduled assessments of antenatal malaria infection by PCR. Children were evaluated at 12, 18, and/or 24 months of age with cognitive tests previously validated in Malawi: the Malawi Developmental Assessment Tool (MDAT) and the MacArthur–Bates Communicative Development Inventories (MCAB-CDI). We assessed the impact of antenatal malaria (*n* [%] positive: 240 [57.3]), placental malaria (*n* [%] positive: 112 [29.6]), and maternal immune activation on neurocognitive development in children. Linear mixed-effects analysis showed that children exposed to antenatal malaria between 33 and 37 weeks gestation had delayed language development across the 2-year follow-up, as measured by MCAB-CDI (adjusted beta estimate [95% CI], −7.53 [−13.04, −2.02], *p =* 0.008). Maternal immune activation, characterized by increased maternal sTNFRII concentration, between 33 and 37 weeks was associated with lower MCAB-CDI language score (adjusted beta estimate [95% CI], −8.57 [−13.09, −4.06], *p <* 0.001). Main limitations of this study include a relatively short length of follow-up and a potential for residual confounding that is characteristic of observational studies.

**Conclusions:**

This mother–baby cohort presents evidence of a relationship between malaria in pregnancy and neurodevelopmental delay in offspring. Malaria in pregnancy may be a modifiable risk factor for neurodevelopmental injury independent of birth weight or prematurity. Successful interventions to prevent malaria during pregnancy may reduce the risk of neurocognitive delay in children.

## Abstract 2

Andrea Weckman and co-workers study associations between children’s neurodevelopmental outcomes and malaria in pregnancy.

## Author summary

**Why was this study done?:**

- Large human cohorts have linked maternal infection during pregnancy (without congenital infection) to an increased risk for neurocognitive deficits and neuropsychiatric disease in exposed children. Excessive maternal immune activation, irrespective of pathogen, is thought to mediate this effect.
- Millions of pregnant women are at risk for malaria infection each year. To our knowledge, the impact of malaria during pregnancy on child neurodevelopment has not been systematically studied in a clinical setting.

**What did the researchers do and find?:**

- We conducted an observational cohort follow-up study nested within a larger randomized controlled trial with detailed records of malaria in pregnancy to examine the impact of malaria in pregnancy on neurocognitive development in exposed children.
- To our knowledge, our data provide the first indication in a clinical cohort that malaria in pregnancy may be a previously unrecognized risk factor for neurodevelopmental delay.
- Furthermore, the association of systemic inflammation with reduced neurocognitive scores highlights a potential mechanistic pathway through which malaria in pregnancy could be affecting fetal neurodevelopment.

**What do these findings mean?:**

- There are millions of children in malaria-endemic regions who are not currently meeting their developmental potential. Malaria in pregnancy may be a modifiable risk factor for neurodevelopmental delay in children exposed in utero.
- Our findings suggest that scaling up efforts to prevent malaria infection could be an effective strategy to protect mother–baby dyads from poor developmental outcomes.
- Larger trials powered to address potential residual confounding, with a longer follow-up period and more diverse neurocognitive and neuropsychiatric tests, are required to confirm and extend these findings.

## Introduction

Early developmental delays are prevalent in low- and middle-income countries (LMICs), where an estimated 1 in 3 preschool-age children do not reach cognitive and socio-emotional milestones [1]. This proportion approaches 1 in 2 in sub-Saharan Africa (44%) [1], and these early developmental delays are proven predictors of worse long-term educational attainment, economic productivity, and psychiatric disease in adulthood [1,2]. Identifying barriers to achieving key developmental milestones for children in LMICs is critical to realizing global equity for all children.

A growing body of evidence links maternal infection during pregnancy to neurocognitive deficits and neuropsychiatric disease in exposed offspring [3,4]. Since congenital infection is not required for this effect, maternal immune activation is thought to be a critical determinant of these neurological outcomes [3,4]. Neurodevelopment is a complex, tightly regulated process involving key components of the immune system (e.g., cytokines and the complement system) as essential non-immune mediators of neurodevelopmental processes including neurogenesis and neuronal migration [3,4]. In sub-Saharan Africa, the majority of pregnancies are at risk for malaria infection in pregnancy [5]. Malaria in pregnancy is associated with severe health consequences for mother and child, including maternal anemia, pregnancy loss, and low birth weight (LBW) due to preterm birth (PTB) and/or fetal growth restriction [6]. In particular, the pathophysiology of *Plasmodium falciparum* malaria infection in pregnancy is driven by the accumulation of parasitized erythrocytes binding to chondroitin sulfate A in the placental intervillous space, where they lead to the recruitment, retention, and activation of mononuclear cells [6]. Malaria infection induces systemic and placenta-localized inflammation [6–9], involving several inflammatory mediators (e.g., TNF, CHI3L1, and CRP) with known roles in neurodevelopment [10–13]. Collectively, these data support the hypothesis that in utero exposure to *P*. *falciparum* malaria in pregnancy could interfere with normal neurodevelopment in children.

Preclinical studies of murine malaria in pregnancy revealed an association between in utero exposure to malaria and developmental deficits in memory and affective-like behavior in offspring that persisted to adulthood (without congenital infection or a LBW phenotype) [14–16]. Furthermore, a case study of premature, dizygotic, placental-malaria-discordant twins reported that the twin with evidence of past placental malaria exhibited worse neurocognitive scores at 12 and 24 months of age than the twin without placental malaria [17]. To date, this single case study is the only reported clinical investigation linking malaria infection during pregnancy with neurodevelopmental outcomes in children. Here, we test the hypothesis that malaria in pregnancy, independent of effects on birth weight and prematurity, dysregulates inflammatory pathways that mediate in utero neurodevelopment, and ultimately causes neurocognitive delay. We tested this hypothesis in a longitudinal neurocognitive assessment of a cohort of 421 children born to women enrolled in a clinical trial of malaria prevention in pregnancy in Malawi.

## Methods

### Parent trial study population and procedures

This study enrolled mother–baby dyads from women enrolled in a previous 3-site, 2-arm randomized superiority trial of prevention of malaria in pregnancy in Malawi [18]. HIV-negative pregnant women were enrolled in the parent trial between July 2011 and March 2013 and randomized to receive intermittent preventative treatment in pregnancy with sulfadoxine–pyrimethamine (IPTp-SP) or intermittent screening and treatment in pregnancy with dihydroartemisinin–piperaquine (ISTp-DP) [18]. At enrollment in the parent trial, medical and obstetric history, as well as demographic and educational information, was collected, and all women were provided with an insecticide-treated bed net. Socioeconomic status was calculated using a principal component analysis (PCA) that considered household assets and characteristics assessed using a locally adapted questionnaire (e.g., type of building materials, source of cooking fuel, source of water, electricity, and household appliances/furniture). Gestational age at enrollment was determined by ultrasound. Depending on gestational age at enrollment, women attended 3 or 4 scheduled antenatal visits, every 4–6 weeks after enrollment. At each visit, malaria was assessed using microscopy and real-time polymerase chain reaction (PCR) [18]. At delivery, birth weight, gestational age, congenital anomalies, and the presence of malaria parasites assessed by placental histopathology and by PCR and microscopy in maternal peripheral, placental, and cord blood were recorded. *P*. *falciparum* parasites were detected in all sample types using a real-time PCR assay targeting the *P*. *falciparum* lactate dehydrogenase gene that detects down to a density of 2 parasites/microliter [19]. Here, we focused on PCR-positive infections due to the more reliable detection of malaria infection by real-time PCR compared to microscopy [20]. Congenital anomalies were further recorded at 7 days and 6 to 8 weeks postnatally. A detailed description of inclusion/exclusion criteria for the parent trial, malaria testing, and treatment courses has previously been published [18].

At each antenatal visit, a maternal plasma sample was collected and stored at −80°C. Inflammatory plasma analyte data for this cohort were derived as described [7]. Briefly, Luminex and ELISA (R&D Systems, Minneapolis, MN) were used to measure inflammatory factors in maternal plasma, including chitinase-3-like protein 1 (CHI3L1), soluble tumor necrosis factor receptor II (sTNFRII), and C-reactive protein (CRP). Plasma markers were measured at 3 gestational windows with corresponding malaria in pregnancy data (14 to 23 weeks gestation, >28 to 33 weeks gestation, and >33 to 37 weeks gestation) [7]. Plasma markers were not measured between 23 and 28 weeks gestation [7].

The parent trial was registered with the Pan African Clinical Trials Registry (PACTR201103000280319) and ISRCTN Registry (ISRCTN69800930). Ethical approval for the current study was obtained from the Malawian College of Medicine Research and Ethics Committee (COMREC reference number: P.08/13/1477), the University Health Network Research Ethics Board (REB number: 13-6741-AE), and the University of Liverpool (IREC number: RETH000693). Written informed consent for participation of infants was obtained from caregivers.

### Neurodevelopmental follow-up sub-study (PAMaNeD)

#### Study population

**Fig 1:**
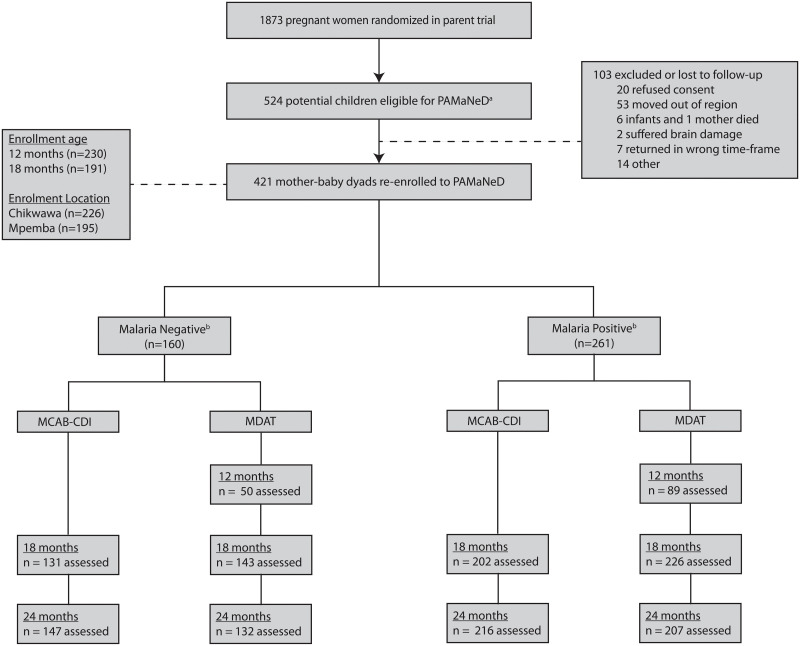
Flow chart for enrollment of mother–baby dyads from parent trial into the PAMaNeD cohort. ^a^Eligibility potential defined by the age of the child (i.e., had to be between 12 and 18 months of age during the study enrollment period). ^b^Malaria negative defined as peripheral PCR negative throughout pregnancy and negative for placental malaria (by histology and placental PCR). Malaria positive defined as any peripheral PCR-positive malaria and/or positive for placental malaria (by histology and/or placental PCR). MCAB-CDI, MacArthur–Bates Communicative Development Inventories; MDAT, Malawi Developmental Assessment Tool.

Between April 2014 and April 2015, children born in both treatment arms of the parent trial were subsequently enrolled in the PAMaNeD cohort study (The Effect of Pregnancy Associated Malaria on Early Childhood Neurocognitive Development: An Observational Birth Cohort Study). Enrollment in the PAMaNeD cohort study began after women had completed their involvement in the parent trial (the final follow-up visit in the parent trial was at 6–8 weeks post-delivery). To mitigate selection bias, every attempt was made to trace, contact, and re-consent all eligible mother–baby pairs (*n =* 524; Fig 1). Mother–baby dyads were eligible for inclusion in PAMaNeD if the infant was between 12 and 18 months in chronological age, and the caretakers were willing to complete study follow-up. Children with major congenital abnormalities, as determined in the parent trial (19/1,722, 1.1% of babies born in parent trial), were excluded. No children in this cohort had neonatal jaundice (405/421 [96.2%] with available data for neonatal jaundice). At enrollment, a detailed history of childhood illness from the final infant follow-up visit in the parent trial (i.e., 6–8 weeks post-delivery) was recorded from the child’s health passport, which is a document provided to each child at birth and is required to seek medical attention from health facilities.

Children were seen at 12, 18, and 24 months of age. At each clinic visit, a clinical history and physical exam were conducted, and any illnesses since the last visit were recorded and corroborated using the child’s health passport. Bed net use was recorded at each visit, and the majority of children (12 months: 140/140 [100%]; 18 months: 366/382 [95.8%]; 24 months: 344/356 [96.6%]) were reported to have slept beneath a bed net the previous night. If the child was ill at the time of assessment, the visit was rescheduled for when they were well. Mothers were encouraged to bring infants with any signs or symptoms of illness to the study clinic between scheduled visits.

#### Procedures

At 12, 18, and 24 months of age, the child’s home environment was assessed using the Family Care Indicator (FCI) questionnaire. The FCI assesses the ability of the household to meet the physical, mental, and social needs of the child and has been adapted and validated for use in Malawi [21]. On the same day, a set of 4 validated tests (described below) were directly administered, observed, and scored by trained personnel to assess child neurodevelopment. Reliability of testers was checked before the start of the trial, and quarterly during the trial, by comparing results from testers to those of a reference trainer (>10 years of experience) (S1 Text). Methods of assessment were the same across exposure groups, and maternal malaria status was unknown to the testers.

#### Malawi Developmental Assessment Tool (MDAT)

At 12, 18, and 24 months, the MDAT was used to assess age-specific neurocognitive development [22]. The MDAT is comprised of 138 items and measures a child’s development across 4 domains: gross motor (36 items), fine motor (36 items), language (36 items), and social skills (30 items). The MDAT is reported as a total score (a combination of items achieved across all domains), as well as the total number of items achieved per domain.

#### MacArthur–Bates Communicative Development Inventories (MCAB-CDI)

At 18 and 24 months, the MCAB-CDI was used to assess language development [23]. The MCAB-CDI is a maternally reported checklist (administered by trained personnel) containing 100 vocabulary items, 6 gesture items, and 5 grammatical items. The MCAB-CDI score is reported as the total number of checklist items correct. This tool has been adapted for use in Malawi, and is a well-validated, reliable measure and predictor of child development that provides a more comprehensive assessment of language than the MDAT [24,25].

#### A-not-B test and delayed inhibition test

At 18 and 24 months, children were assessed using the A-not-B test [26] and delayed inhibition test [27]. These methods and data are presented in S1 Text and S1 Table, rather than the main text, due to the uninformative test results, consistent with other recently published studies (e.g., high rates of non-participation, data skewed towards perfect scores, and no association with known covariates of neurodevelopment including socioeconomic status and FCI) [28].

This study is reported according to STROBE guidelines (S1 STROBE Checklist).

### Outcomes and statistical analyses

The primary outcomes presented here are total MCAB-CDI and total MDAT scores through repeated measures at 12, 18, and 24 months. A subanalysis was performed on the subdomains of the MDAT (i.e., gross motor, fine motor, language, and social skills). Malaria infection was analyzed by 2 exposure definitions: (1) placental malaria, defined as positive placental histology (including chronic and acute active placental malaria and past placental malaria by histology) or positive placental PCR at delivery, and (2) antenatal malaria, defined as PCR-confirmed malaria infection during pregnancy at any scheduled or unscheduled visit including enrollment and delivery (peripheral). The latter was also stratified by specific gestational age windows (14 to 23 weeks gestation, >23 to 28 weeks gestation, >28 to 33 weeks gestation, and >33 to 37 weeks gestation). There were 91 (21.6%) women who met the definition for both antenatal malaria and placental malaria; however, the 2 exposure groups are not directly compared and do not overlap within a single analysis. A detailed breakdown of the characteristics of malaria infections in this cohort (e.g., average number of PCR infections per woman and proportion of active versus past placental malaria) is provided in S2 Table. Due to the dynamic nature of neurodevelopment and inflammatory and/or neurodevelopmental mediators that could be disrupted by antenatal malaria, we analyzed the impact of both malaria infection and inflammatory mediators on neurodevelopment according to gestational age at the time of the insult. Here, to link the timing of malaria infections with maternal analyte analysis, gestational age bins were chosen to coincide with the windows previously used to measure inflammatory mediators across pregnancy [7]. In that study (of which our study population is a subset), malaria infection was associated with increases in these inflammatory mediators [7]; these associations have not been presented here to prevent double-reporting. There was no association between PCR-positive cord blood (9.8% [35/356]) and neurocognitive outcomes (S3 Table); therefore, children with PCR-positive cord blood were included in the cohort.

Statistical analysis was performed using R version 3.5.1 (R Foundation for Statistical Computing, Vienna, Austria). The prespecified statistical analysis is described in the study protocol (S1 Protocol). In addition to the prespecified analyses, we used linear mixed-effects (LME) modeling to estimate the impact of malaria in pregnancy on longitudinal, repeated measurements of a child’s neurocognitive scores across time. We compared baseline population characteristics using the Pearson chi-squared (categorical) or Wilcoxon rank-sum test (continuous). We used unadjusted ordinary least squares regression to estimate the baseline effect of antenatal and placental malaria on neurocognitive scores in children at 12, 18, and 24 months of age.

To examine the impact of maternal malaria infection and inflammation on longitudinal neurodevelopment, we built LME models using the lme4 package in R [29]. LME modeling allows for longitudinal analysis of repeated measures data (multiple observations per child across time), while accounting for heterogeneity in baseline scores and within-individual correlation between repeated measures. Longitudinal scores (repeated measures at 12, 18, and 24 months of age for MDAT, or 18 and 24 months for MCAB-CDI) were modeled as the outcome variable for each neurocognitive assessment. Each time point for which a child had a recorded score was included as a separate but related observation (repeated measure) in the model. Confounders were considered a priori based on documented associations with malaria in pregnancy or early developmental outcomes, and were included in the model when adequate data were available. All mixed-effects models included maternal age, socioeconomic status (continuous), FCI (a measure of home environment), birth weight (continuous), age of the child at assessment (corrected for prematurity), number of recorded child malaria infections, and sex of the child as fixed effects (confounders), as well as a by-participant random intercept. Adjusted beta estimates (95% CIs) for our exposure variables of interest are reported in the Results: These represent the difference in raw neurocognitive score with a 1-unit change in our variable of interest (e.g., malaria negative versus malaria positive or a 1-unit increase in analyte concentration), holding other fixed effects constant. There were no significant interaction effects between any variables of interest (e.g., maternal malaria status and analyte data) and child’s assessment age, indicating that effects did not significantly vary by visit. A restricted cubic spline of age at assessment (with 3 knots) was used to further capture variation across time, where appropriate. Maternal age and gravidity were strongly correlated; therefore, only maternal age was included in models, to avoid multicollinearity. Age at assessment was corrected for gestational age at delivery. There were no differences in baseline characteristics or longitudinal neurocognitive scores between treatment arms (S4 Table); however, treatment arm was included as a fixed effect to account for parent trial design. For each neurocognitive assessment, we built a null model containing the fixed and random effects defined above. We then added the exposure variable of interest (antenatal malaria, placental malaria, or inflammatory analyte concentration) as a main effect and compared models to assess the impact of exposure on model fit (using the likelihood ratio test). The Akaike information criterion (AIC) is reported as a measure of model fit. Analyte data were log-transformed. Missing data were excluded from analyses (S5 Table).

*p*-Values were adjusted for multiple comparisons using the Holm–Bonferroni method (S1 Text). Uncorrected *p*-values are presented. *p*-Values considered significant after correction for multiple comparisons using the Holm–Bonferroni method are marked with an asterisk in the figures.

## Results

### Enrollment, follow-up, and baseline characteristics

During the enrollment period, 524 children born to women in the parent trial were between 12 and 18 months of age and potentially eligible for inclusion. Of those, 421 were enrolled (Fig 1): 230 (54.6%) at 12 months and 191 (45.4%) at 18 months. Overall, 57.3% (240/419) and 29.6% (112/378) of children were born to pregnancies that had evidence of antenatal malaria and placental malaria, respectively (Table 1). Malaria infection during pregnancy was more common in primigravidae and was associated with lower maternal age, lower socioeconomic status, and lower maternal hemoglobin level at enrollment (Table 1). There was no difference between groups in the total number of child infections recorded in the health passport; however, children born to mothers with malaria during pregnancy were more likely to be parasitemic during the study period (Table 1). As malaria infection in childhood is associated with neurocognitive impairment [30], we included malaria infection as a fixed effect in our multivariable models.

**Table 1:** Baseline characteristics of PAMaNeD mother–baby dyads overall and by malaria status in pregnancy.

Characteristic	Overall	Malaria negative^a^	Malaria positive^a^	*p*-Value^b^
*n* (%)	421 (100)	160 (38.0)	261 (62.0)	
**Baseline maternal characteristics**				
Maternal age (years)	21 [19, 25]	23 [20, 27]	20 [18, 23]	<0.001
Gestational age at enrollment (weeks)	19.7 [17.9, 22.1]	20.4 [18.3, 22.5]	19.6 [17.7, 21.9]	0.059
Socioeconomic status (tertile)				0.012
1	125 (29.8)	36 (22.6)	89 (34.2)	
2	145 (34.6)	54 (34.0)	91 (35.0)	
3	149 (35.6)	69 (43.4)	80 (30.8)	
Hemoglobin at enrollment (g/dL)	10.9 [9.9, 12.0]	11.3 [10.3, 12.3]	10.7 [9.7, 11.8]	<0.001
Primigravid	277 (65.8)	40 (25.0)	104 (39.8)	0.003
Maternal education status (tertile)				0.911
1	125 (29.8)	46 (28.9)	79 (30.4)	
2	229 (54.7)	87 (54.7)	142 (54.6)	
3	65 (15.5)	26 (16.4)	39 (15.0)	
Malaria status^c^				
Negative	160 (38.0)			
Antenatal malaria	240 (57.3)			
Placental malaria	112 (29.6)			
**Perinatal and child characteristics**				
Gestational age at delivery (weeks)	38.7 [37.4, 39.9]	38.7 [37.6, 39.9]	38.6 [37.3, 39.9]	0.253
Birth weight (kg)	3.0 [2.7, 3.2]	3.0 [2.8, 3.3]	3.0 [2.7, 3.2]	0.033
Sex				0.123
Male	211 (50.1)	72 (45.0)	139 (53.3)	
Female	210 (49.9)	88 (55.0)	122 (46.7)	
Preterm birth (<37 weeks gestation)	71 (16.9)	22 (13.8)	49 (18.8)	0.229
Low birth weight (<2.5 kg)	28 (6.8)	8 (5.2)	20 (7.8)	0.409
Small for gestational age	33 (8.1)	14 (9.1)	19 (7.5)	0.687
Child infections (all)^d^				0.443
No infections	65 (15.6)	25 (15.6)	40 (15.6)	
≤2 infections	101 (24.2)	44 (27.5)	55 (22.2)	
>2 infections	251 (60.2)	91 (56.9)	160 (62.3)	
Child malaria infections^e^				0.008
No infections	175 (42.1)	75 (46.9)	100 (39.1)	
1 infection	113 (27.2)	50 (31.3)	63 (24.6)	
>1 infection	128 (30.8)	35 (21.9)	93 (36.3)	

Data given as *n* (%) or median [interquartile range] of women with existing data for that variable. Proportion with missing data presented in S5 Table.

^a^Malaria negative defined as peripheral PCR negative throughout pregnancy and negative placental malaria (by histology and placental PCR). Malaria positive defined as any peripheral PCR-positive malaria and/or positive placental malaria (by histology and/or placental PCR).

^b^*p*-Value of chi-squared or Mann–Whitney U test comparing malaria negative and malaria positive.

^c^*n =* 91 women were positive for both antenatal malaria and placental malaria, which accounts for *n* > 421 for this variable. Those women were only counted once in the malaria positive column.

^d^Number of infections (including malaria) reported in child’s health passport up to 24 months.

^e^Number of malaria infections reported in child’s health passport up to 24 months.

### Neurodevelopment in children born to mothers with placental malaria or PCR-confirmed malaria during pregnancy

**Fig 2:**
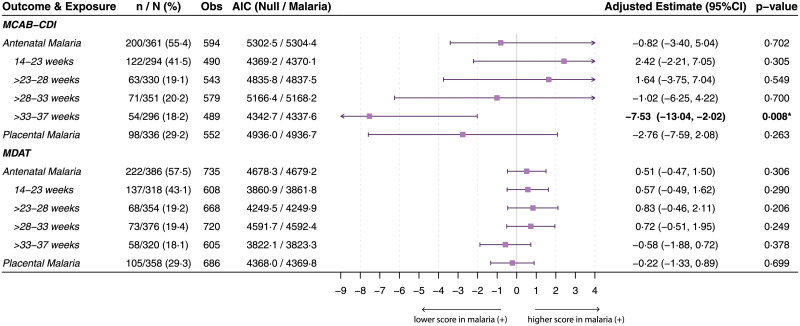
Longitudinal total MCAB-CDI and total MDAT scores in children by antenatal malaria exposure. Results of linear mixed-effects models for repeated neurocognitive score measures over time (12, 18, and 24 months of age for MDAT; 18 and 24 months of age for MCAB-CDI) by maternal malaria status. Malaria exposures defined as antenatal malaria (peripheral PCR-confirmed malaria at any point during pregnancy) or placental malaria. The former is stratified by gestational age (weeks) at time of PCR-positive malaria infection. *n*/*N* (%): malaria-exposed children as percent of total mother–baby dyads (*N*) included in the model (i.e., with existing data for both the respective neurocognitive score and malaria variable). Obs: number of observations (scores) included in each model. Possible range of neurocognitive scores for MCAB-CDI (min/max scores in this cohort across all visits: 0–98) and total MDAT (min/max scores in this cohort across all visits: 42–104). AIC values (parameter of model fit), adjusted beta estimates (difference in raw neurocognitive score between malaria-negative and malaria-positive women, holding other fixed effects constant) with 95% CIs, and likelihood ratio test results (*p*-values) are presented. All models adjusted for maternal age, maternal socioeconomic status, treatment arm, Family Care Indicator score, birth weight, corrected age of child at assessment, number of childhood malaria infections, and sex of child as fixed effects, and a by-participant intercept as a random effect. *p*-Value determined by likelihood ratio test comparing model with malaria exposure variable to null model (without malaria exposure variable). Uncorrected *p*-values are presented; 1 association remained statistically significant after adjustment for multiple comparisons according to the Holm–Bonferroni method (*n =* 6 malaria exposure comparisons) (in bold and marked by an asterisk). AIC, Akaike information criterion; MCAB-CDI, MacArthur–Bates Communicative Development Inventories; MDAT, Malawi Developmental Assessment Tool.

**Fig 3:**
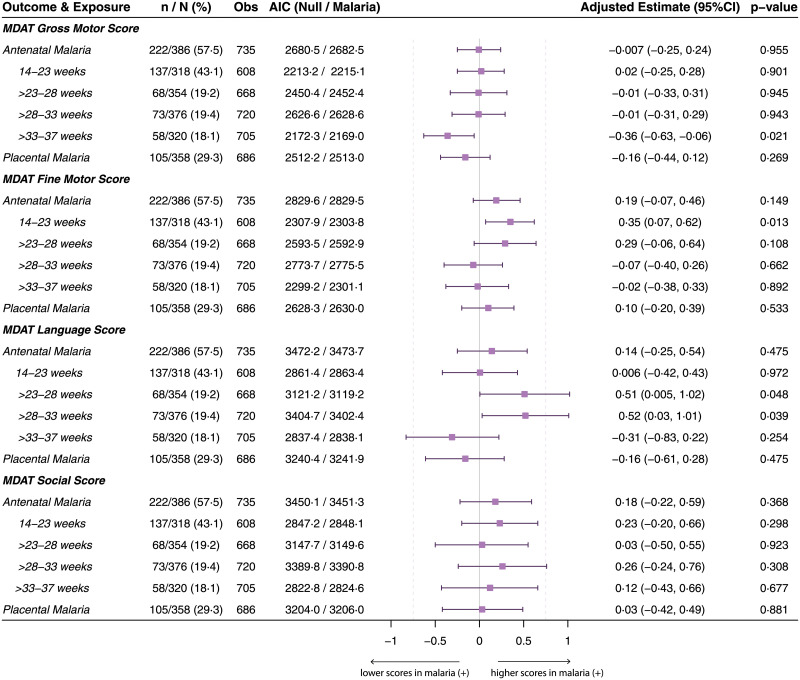
Longitudinal MDAT subdomain scores in children by antenatal malaria exposure. Results of linear mixed-effects models for repeated neurocognitive score measures over time (12, 18, and 24 months of age) by maternal malaria status. Malaria exposures defined as antenatal malaria (peripheral PCR-confirmed malaria at any point during pregnancy) or placental malaria. The former is stratified by gestational age (weeks) at time of PCR-positive malaria infection. *n*/*N* (%): malaria-exposed children as percent of total mother–baby dyads (*N*) included in the model (i.e., with existing data for both the respective neurocognitive score and malaria variable). Obs: number of observations (scores) included in each model. Possible range of MDAT subdomain scores (minimum/maximum scores in this cohort across all visits): gross motor, 12–27; fine motor, 11–29; language, 1–27; and social, 11–33. AIC values (parameter of model fit), adjusted beta estimates (difference in raw neurocognitive score between malaria negative and malaria positive, holding other fixed effects constant) with 95% CIs, and likelihood ratio test results (*p*-values) are presented. All models adjusted for maternal age, maternal socioeconomic status, treatment arm, Family Care Indicator score, birth weight, corrected age of child at assessment, number of childhood malaria infections, and sex of child as fixed effects, and a by-participant intercept as a random effect. *p*-Value determined by likelihood ratio test comparing model with malaria exposure variable to null model (without malaria exposure variable). Uncorrected *p*-values are presented; none of the associations remained statistically significant after adjustment for multiple comparisons according to the Holm–Bonferroni method (*n =* 6 malaria exposure comparisons). AIC, Akaike information criterion; MDAT, Malawi Developmental Assessment Tool.

Raw unadjusted neurocognitive scores at each assessment (12, 18, and 24 months of age) stratified by maternal malaria status are presented in S1 and S6 Tables. Multivariable analysis of the impact of the “any malaria” exposure variable (i.e., any antenatal malaria or placental malaria) on longitudinal neurocognitive scores in children did not show differences (Figs 2 and 3). However, when antenatal malaria was stratified by gestational age at infection, longitudinal language development scores measured by MCAB-CDI were lower in children born to mothers with PCR-positive malaria between 33 and 37 weeks gestation (adjusted beta estimate [95%CI], −7.53 [−13.04, −2.02], *p =* 0.008), compared to children born to mothers who were PCR negative for malaria in this window, even after correcting for multiple comparisons (Fig 2). Stratifying maternal-malaria-associated differences by age at assessment showed that differences in MCAB-CDI score emerged at 18 months of age and persisted up to at least 24 months (S6 Table).

### Maternal immune activation is associated with altered neurodevelopmental trajectories in exposed children

**Fig 4:**
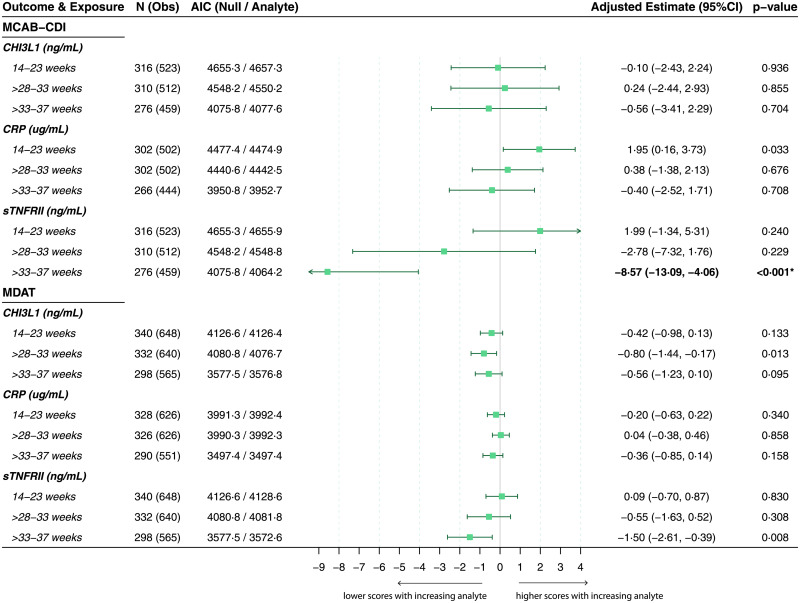
Longitudinal total MCAB-CDI and total MDAT scores in children by maternal inflammatory mediator exposure. Results of linear mixed-effects models for repeated neurocognitive score measures over time (12, 18, and 24 months of age for MDAT; 18 and 24 months for MCAB-CDI) by maternal immune activation. Maternal immune activation defined by inflammatory analyte concentrations by gestational age (in weeks) at time of sample acquisition. *N* represents the total number of children who had both a neurocognitive score and a corresponding maternal analyte measurement at the respective gestational age, and Obs represents the number of observations (scores) included in each model. Possible range of neurocognitive scores for MCAB-CDI (minimum/maximum scores in this cohort across all visits) was 0–98, and for total MDAT (minimum/maximum scores in this cohort across all visits) was 42–104. AIC values (parameter of model fit), adjusted beta estimates (change in raw neurocognitive score for a 1-unit increase in analyte, holding other fixed effects constant) with 95% CIs, and likelihood ratio test results (*p*-values) are presented. All models adjusted for maternal age, maternal socioeconomic status, treatment arm, Family Care Indicator score, birth weight, corrected age of child at assessment, number of childhood malaria infections, and sex of child as fixed effects, and a by-participant intercept as a random effect. *p*-Value determined by likelihood ratio test comparing model with analyte to null model (without analyte). Analyte measurements were log-transformed. Uncorrected *p*-values are presented; 1 association remained statistically significant after adjustment for multiple comparisons according to the Holm–Bonferroni method (4 analyte measurements × 3 analytes; *n =* 12 comparisons) (in bold and marked by an asterisk). AIC, Akaike information criterion; CHI3L1, chitinase-3-like protein 1; CRP, C-reactive protein; MCAB-CDI, MacArthur–Bates Communicative Development Inventories; MDAT, Malawi Developmental Assessment Tool; sTNFRII, soluble tumor necrosis factor receptor II.

**Fig 5:**
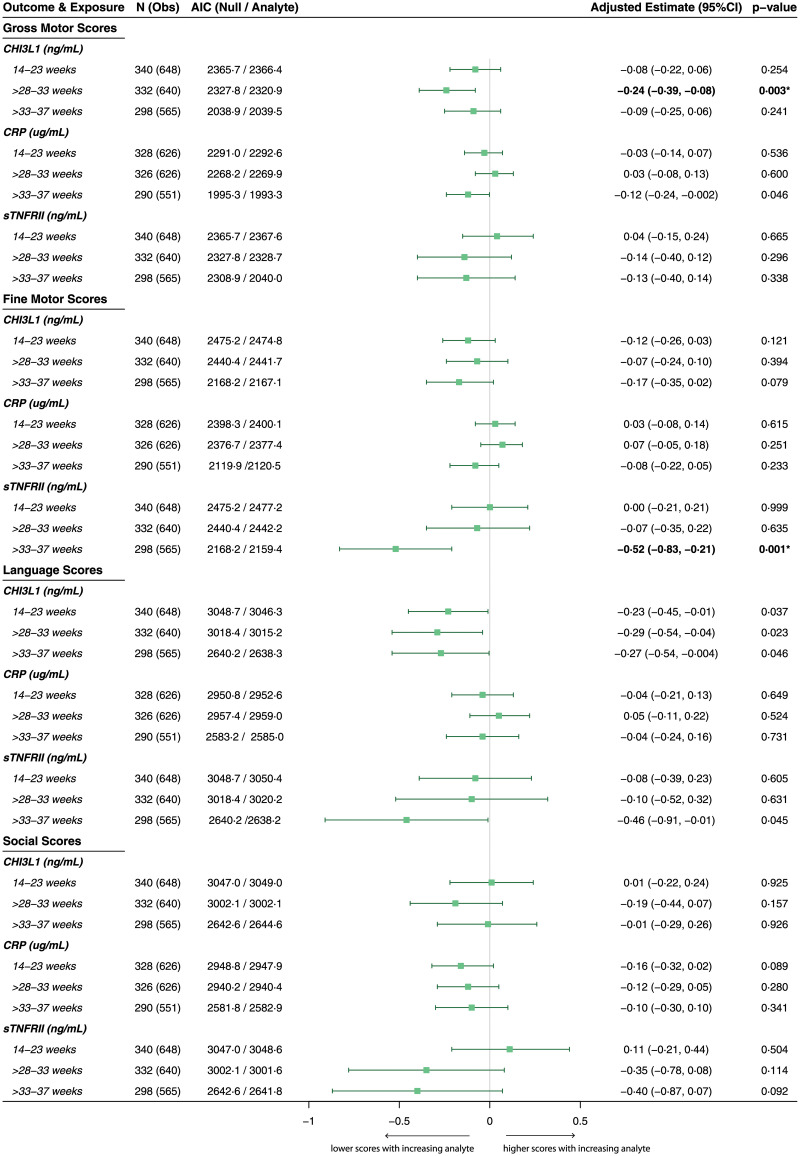
Longitudinal MDAT subdomain scores in children by maternal inflammatory mediator exposure. Results of linear mixed-effects models for repeated neurocognitive score measures over time (12, 18, and 24 months) by maternal immune activation. Maternal immune activation defined by inflammatory analyte concentrations by gestational age (in weeks) at time of sample acquisition. *N* represents the total number of children who had both a neurocognitive score and a corresponding maternal analyte measurement at the respective gestational age, and Obs represents the number of observations (scores) included in each model. Possible range of MDAT subdomain scores (minimum/maximum scores in this cohort across all visits): gross motor, 12–27; fine motor, 11–29; language, 1–27; social, 11–33. AIC values (parameter of model fit), adjusted beta estimates (change in raw neurocognitive score for a 1-unit increase in analyte, holding other fixed effects constant) with 95% CIs, and likelihood ratio test results (*p-*values) are presented. All models adjusted for maternal age, maternal socioeconomic status, treatment arm, Family Care Indicator score, birth weight, corrected age of child at assessment, number of childhood malaria infections, and sex of child as fixed effects, and a by-participant intercept as a random effect. *p*-Value determined by likelihood ratio test comparing model with analyte to null model (without analyte). Analyte measurements were log-transformed. Uncorrected *p*-values are presented; two associations remained statistically significant after adjustment for multiple comparisons according to the Holm–Bonferroni method (4 analyte measurements × 3 analytes; *n =* 12 comparisons) (in bold and marked by an asterisk). AIC, Akaike information criterion; CHI3L1, chitinase-3-like protein 1; CRP, C-reactive protein; MDAT, Malawi Developmental Assessment Tool; sTNFRII, soluble tumor necrosis factor receptor II.

After correcting for multiple comparisons, increased maternal sTNFRII between 33 and 37 weeks gestation was also associated with lower MCAB-CDI score (adjusted beta estimate [95%CI], −8.57 [−13.09, −4.06], *p <* 0.001) (Fig 4) and lower MDAT fine motor skill score (adjusted beta estimate [95%CI], −0.52 [−0.83, −0.21], *p =* 0.001) (Figs 4 and 5). Higher maternal CHI3L1 between 28 and 33 weeks was associated with reduced MDAT gross motor skill score (adjusted beta estimate [95%CI], −0.24 [−0.39, −0.08], *p =* 0.003) (Fig 5).

## Discussion

Here we assessed longitudinal neurodevelopment up to 24 months of age, in a cohort of 421 Malawian children born to mothers with well-characterized antenatal and placental malaria. After controlling for confounding factors, the strongest association in our data linked PCR-positive malaria late in pregnancy (33–37 weeks gestation) with impaired language development up to 2 years of age. Furthermore, analysis of inflammatory mediators supports the hypothesis that this association is driven by maternal immune activation, as increased maternal plasma concentration of sTNFRII between 33 and 37 weeks gestation was associated with reduced language score in exposed children. These findings provide evidence that malaria in pregnancy may be an underrecognized risk factor for developmental delay in malaria-endemic regions.

The profile of neurodevelopmental delay associated with malaria in pregnancy and maternal immune activation (e.g., motor and language deficits) in our cohort is consistent with previous findings for other maternal infections in women living in LMICs [31–34]. Mechanistically, maternal immune activation induced by infection is hypothesized to be an important contributor to neurological sequelae in exposed children [3,4]. In the parent trial, malaria during pregnancy was associated with elevation of inflammatory mediators including sTNFRII, CRP, and CHI3L1 across pregnancy [7]. The data in our study suggest that this malaria-mediated inflammation (i.e., increased sTNFRII and CHI3L1) may be associated with delayed neurocognitive development in exposed children. CHI3L1 has been implicated in the pathogenesis of neurological disorders including psychiatric disease and multiple sclerosis, and as a modulator of neuroinflammation [35,36]. CHI3L1 is expressed in the developing human brain beginning early in gestation (first trimester), with possible roles in the formation of the blood–brain barrier, astrogliogenesis, and astrocyte migration [10,11]. During development, TNF signaling has roles in synaptic plasticity, cortical dendrite development, and sympathetic axon growth and innervation [12,37]. High circulating maternal TNF has been associated with increased risk of schizophrenia and severity of autism spectrum disorder in individuals exposed in utero [3]. While our data support a role for inflammation in malaria-driven disruptions to neurodevelopment, these markers do not provide a comprehensive representation of immune activation by malaria during pregnancy. Many cytokines and chemokines (e.g., IL-6 and the complement system) have been associated with the neurocognitive sequelae of maternal infection [3,4], and future studies should expand the panel of inflammatory markers that could link malaria in pregnancy with neurodevelopment.

Here, antenatal-malaria-associated and sTNFRII-associated decreases in MCAB-CDI language development scores represented the largest, and most clinically relevant, effect size. Maternal sTNFRII and antenatal malaria between 33 and 37 weeks gestation were both associated with significantly lower scores in language development in exposed children. There were some differences in MDAT scores by maternal malaria status, but these did not remain significant after correction for multiple comparisons and lacked clinical relevance given the modest effect sizes (<1 item difference on a 138-item assessment tool). Conversely, the effect sizes for language development using the MCAB-CDI are significant for under-2 child development [38], and greater than previous studies in similar populations using the same tool [28,39]. From a technical perspective, the discrepancy in differences seen with the MCAB-CDI score but not with the MDAT language subscore could be explained by the relative sensitivity of the tests. The MCAB-CDI and MDAT language subdomain scores were correlated (Spearman correlation, rho = 0.65, *p <* 0.001), in agreement with previously published comparisons [25]. However, the MCAB-CDI assesses 111 checklist items directly related to language, whereas language development is a 36-item subsection on the MDAT, making the MCAB-CDI more comprehensive for language assessment and more sensitive to potential differences, as our data show.

The impact of the timing of maternal infection on neurodevelopment remains largely unknown. One preclinical study in a murine model of maternal immune activation revealed distinct profiles of neurological sequelae depending on the gestational timing of insult [40]. Our data indicate that malaria infection late in pregnancy (33–37 weeks gestation; late third trimester) may be detrimental for language development. Mechanistically, this finding is supported by timelines of neurodevelopment [13,41]. The third trimester is a critical period for the development of functional neural circuitry, and maternal immune activation during the third trimester impairs neural network connectivity [13,41]. Neural circuits for language processing are complex, and these networks begin forming in utero, with established foundations for the neurobiological basis of language by birth [41,42]. Therefore, insults like malaria-induced maternal immune activation that interfere with third-trimester neural network building processes (e.g., neuronal migration, dendritic arborization, synaptogenesis and synaptic pruning, and myelination) could result in language deficits in children, as we observed. As disruptions in early-life language development are associated with long-term cognitive deficits and increased risk for psychiatric comorbidities, this finding may have long-term implications for children exposed to malaria late in gestation. However, this does not exclude an impact of malaria early in pregnancy on fetal neurodevelopment. Other studies have shown an association between first-trimester infection and cognitive and psychiatric outcomes in offspring, and it is possible that the tests used here may not be sensitive enough or appropriate (e.g., autism spectrum disorder has been associated with first-trimester infections) [43,44] to detect the gestational-timing-dependent profile of neurological sequelae associated with malaria infection early in pregnancy.

Even in the context of this relatively healthy cohort, malaria during pregnancy was associated with neurodevelopmental deficits in language after controlling for birth weight, gestational age at delivery, and sociodemographic factors that impact child development. Malaria in pregnancy is associated with adverse birth outcomes, including LBW due to small for gestational age (SGA) and/or PTB [6]. LBW and PTB are well-characterized risk factors for poor long-term neurodevelopmental outcomes. Compared to the parent study, the PAMaNeD cohort had fewer cases of malaria and fewer adverse birth outcomes (PTB, LBW, and SGA) (S7 Table). As birth outcomes were better in the PAMaNeD cohort compared to the parent trial population, and children with congenital abnormalities or brain damage were excluded, these data indicate that the scope of antenatal-malaria-related neurodevelopmental deficits on a population level may be greater than the estimates in this cohort. Furthermore, seminal work on maternal infection and neurodevelopment revealed an increased risk of psychiatric outcomes, including schizophrenia, autism spectrum disorder, and depression [3,43–45]. Mental illness represents a leading cause of disability-adjusted life years (DALYs) globally [46], exerting a huge social and financial burden in LMICs [47], many of which are malaria-endemic. Future studies are needed to extend the follow-up of children exposed to antenatal malaria in a larger study population, who may also be at increased risk of neuropsychiatric disorders.

Strengths of this study include a comprehensive evaluation of the association between malaria infection during pregnancy and neurodevelopment in children nested within the context of a previously conducted, rigorous prospective randomized controlled trial. Malaria was repeatedly assessed across pregnancy using PCR, which enabled us to evaluate how the timing of malaria infection during pregnancy impacted developmental outcomes. Our findings were strengthened by longitudinal assessment of inflammatory analytes across pregnancy, which allowed us to examine potential mechanisms linking malaria during pregnancy to neurodevelopmental outcomes. Limitations of this study include the limited length of follow-up, the potential for residual confounding, and the number of comparisons. However, *p*-values were adjusted for the number of comparisons to mitigate the effect of multiple comparisons on the type I error rate. As an observational study where the exposure variable cannot be allocated at random (e.g., presence of antenatal or placental malaria), the study was prone to bias. This bias was mitigated by measurement and consideration of as many potential confounders as possible (e.g., socioeconomic status, birth weight, FCI, and child malaria infections); however, some residual confounding may remain, and larger trials powered to address residual confounding are necessary to confirm and extend these findings. Residual confounders that are associated with neurodevelopment in LMICs but that we were unable to measure could include other maternal or child infections (e.g., helminth infections [48–50]), childhood anemia or iron deficiency [51], and anthropometric characteristics beyond birth weight. Malaria exposure in utero may modify a child’s risk of malaria infection, which is known to affect development [30]. Here, we adjusted for any symptomatic malaria in childhood, but it remains possible that unmeasured asymptomatic infections could also play a role. The implications in a population with high rates of malaria in pregnancy remain the same, however: Better malaria protection during pregnancy and infancy is likely to improve child development via several converging pathways. There may also have been potential biases in re-enrollment, as it is possible that mothers of children with moderate to severe disability did not re-enroll for a variety of reasons (e.g., stigma, resources, or death of the child). Additional studies to evaluate the long-term impact of malaria during pregnancy would be beneficial, as tests become more sensitive to detect differences in specific developmental domains. It is possible that more sensitive and objective neurobiological tests, including electroencephalogram and eye tracking, could enhance the information gained from this study; however, these techniques are challenging and not routinely administered in LMICs. This population enrolled women who were HIV negative. Maternal HIV infection is associated with worse neurodevelopmental outcomes in HIV-exposed infected children and HIV-exposed uninfected children compared to non-exposed children [33]. Given the prevalence of malaria and HIV coinfection in LMICs, this may limit the generalizability of our findings, and studies to evaluate whether malaria and HIV interact to drive worse outcomes are needed.

In Malawi, an estimated 40% of children have low early childhood development scores [1], similar to many resource-limited and malaria-endemic regions worldwide [1]. The data presented here provide clinical evidence that, in malaria-endemic regions, antenatal malaria may be an unrecognized but modifiable risk factor for neurocognitive deficits in children. The notion that antenatal malaria primes children for neurocognitive delay represents a paradigm shift in our understanding of risk factors that potentially contribute to millions of children not meeting their developmental potential. Our data suggest that scaling prevention efforts for malaria infection during pregnancy could be a far-reaching strategy to simultaneously reduce maternal and child morbidity and mortality and enhance the ability of children in malaria-endemic regions to meet their developmental potential.

## Supporting information

S1 Data Anonymized dataset, wide format. (XLSX)

S1 Protocol PAMaNeD study protocol P.08/13/1447. (PDF)

S1 STROBE Checklist STROBE checklist for the reporting of cohort studies. (DOC)

S1 Table Raw A-not-B and delayed inhibition neurocognitive scores at 18 and 24 months by maternal malaria status during pregnancy. (DOCX)

S2 Table Detailed breakdown of maternal malaria infections in cohort. (DOCX)

S3 Table MDAT and MCAB-CDI at 12, 18, and 24 months by cord-blood PCR malaria status. (DOCX)

S4 Table Descriptive characteristics of the PAMaNeD study population by parent trial treatment arm. (DOCX)

S5 Table Summary of missing and available data. (DOCX)

S6 Table Raw MCAB-CDI and MDAT scores (mean, SD) at 12, 18, and 24 months by maternal malaria status during pregnancy. (DOCX)

S7 Table Descriptive characteristics of the PAMaNeD study cohort versus the parent trial cohort. (DOCX)

S1 Text Supplementary methods. (DOCX)

## Abbreviations

AIC: Akaike information criterion
FCI: Family Care Indicator
LBW: low birth weight
LME: linear mixed-effects
LMICs: low- and middle-income countries
MCAB-CDI: MacArthur–Bates Communicative Development Inventories
MDAT: Malawi Developmental Assessment Tool
PTB: preterm birth
SGA: small for gestational age
