# Supplementary material for: Neurocognitive outcomes in Malawian children exposed to malaria during pregnancy: An observational birth cohort study
Source: PLoS Med. 2021 Sep 28;18(9):e1003701. doi: 10.1371/journal.pmed.1003701 (PMC8478258; doi:10.1371/journal.pmed.1003701)
Supplement: S1 Protocol — (PDF) [file pmed.1003701.s002.pdf]

UNIVERSITY OF MALAŴI

Principal

K. M . Maleta MBBS, PhD

Our Ref.:

Your Ref.: P.05/10/938

College of Medicine
Private Bag 360
Chichiri
Blantyre 3
Malawi
Telephone: 877 245
877 291
Fax: 874 700
Telex: 43744

TO: The Chairperson, COMREC

FROM: Dr Mwayiwawo Madanitsa

DATE: Thursday, 30th January 2014

Submission of Amendment to Research Protocol P.08/13/1447 **"**The Effect of Pregnancy Associated Malaria on Early Childhood Neurocognitive Development: An Observational Birth Cohort Study (PAMaNeD), version 2.0 dated 18th September 2013"

Due to unanticipated delays in disbursement of funding from the study sponsors, based on enrolment of infants at 12 months, an estimated 58% successful re-enrollment rate and an estimated 20% attrition rate, the study would lack adequate power to address the primary research questions (Appendix A). In light of this, the study investigating team has considered to employ a two part enrollment procedure whereby an additional 264 infants will be enrolled at 18 months.

With an estimated 58% successful re-enrollment rate at 12 months, there will still be adequate power to make comparisons of outcomes at 12 months (Appendix A).

I hereby submit an amendment of the above referenced protocol as version 3.0 dated 30th January 2014 in track-changes as well as the earlier approved protocol version 2.0 dated 18th September 2013, for the review of your committee. The amendments have been implemented as follows:

1. Revised consent procedures to include 18 months, page 23.
2. Added 'Enrolment procedures: Statistical considerations', page 26-27.

Please acknowledge in writing receipt of this resubmission. Your cordial and continued support is greatly appreciated.

Sincerely.

Mwayiwawo Madanitsa
PhD Student, Division of Community Health

APPENDIX A: Statistical considerations on amendment of enrolment procedures

1. **Inadequate power at study end based on enrolment of participants only at 12 months of age.**
   Assumptions:
   a. 403 infants for re-enrolment
   b. 58% successful re-enrolment
   c. 30% exposed
   d. 20% attrition rate

```
. sampsi 0.2 0.4, alpha(.05) n1(124) n2(62)

Estimated power for two-sample comparison of proportions

Test Ho: p1 = p2, where p1 is the proportion in population 1
                    and p2 is the proportion in population 2
Assumptions:

         alpha =   0.0500  (two-sided)
            p1 =   0.2000
            p2 =   0.4000
sample size n1 =      124
            n2 =       62
         n2/n1 =     0.50

Estimated power:

         power =   0.7700
```

2. **Adequate power at 12 month enrolment to compare primary outcomes**
   Assumptions:
   a. 403 infants for re-enrolment
   b. 58% successful re-enrolment
   c. 30% exposed

```
. sampsi 0.2 0.4, alpha(.05) n1(155) n2(78)

Estimated power for two-sample comparison of proportions

Test Ho: p1 = p2, where p1 is the proportion in population 1
                    and p2 is the proportion in population 2
Assumptions:

         alpha =   0.0500  (two-sided)
            p1 =   0.2000
            p2 =   0.4000
sample size n1 =      155
            n2 =       78
         n2/n1 =     0.50

Estimated power:

         power =   0.8626
```

Principal

K.M Maleta, MBBS PhD

Our Ref.:

Your Ref.: P.08/13/1477

College of Medicine
Private Bag 360
Chichiri
Blantyre 3
Malawi
Telephone: 01 877 245
01 877 291
Fax: 01 874 700

20th February 2014

Dr. M. Madanitsa
College of Medicine
UNC Project
P/Bag 360
Blantyre 3

Dear Dr Madanitsa,

RE: P.08/13/1477 – The effect of the pregnancy associated malaria on early childhood neurognitive development: an observational birth cohort study version 2.0 dated 18th September 2013

I write to inform you that COMREC reviewed the amendments to the above mentioned study which you submitted for review. I am pleased to inform you that COMREC **approved** the following requests:

1. The revised consent procedures to include 18 months
2. The additional of "Enrolment procedures: statistical considerations

As you proceed with the implementation of your study we would like you to take note that all requirements by the college are followed as indicated on the attached page.

Sincerely,

Approved by
College of Medicine
20 FEB 2014
(COMREC)
Research and Ethics Committee

Dr. C. Dzamalala
For: COMREC CHAIRPERSON

CD/*ck*

The Effect of Pregnancy Associated Malaria on Early Childhood Neurocognitive Development: an Observational Birth Cohort Study (PAMaNeD)

Principal Investigator:

1. Dr Mwayiwawo Madanitsa, MBBS (Community Health Dept, College of Medicine)

Co-Investigators:

2. Dr. Victor Mwapasa, MBBS, MPH, PhD. (Community Health Dept., College of Medicine)
3. Dr Andrea Conroy, BSc, PhD (University of Toronto)
4. Dr Melissa J Gladstone, (MBChB, BSc, MRCP, MRCPCH, MD) (University of Liverpool)
5. Dr. Linda Kalilani-Phiri, MBBS, MPhil, PhD (Community Health Dept, College of Medicine)
6. Dr Doreen Ali, BSC.NEAD, MSC.CEB (National Malaria Control Programme, Ministry of Health)
7. Professor Feiko ter Kuile, MD, PhD (Liverpool School of Tropical Medicine)
8. Professor Kevin Kain, MD, FRCPC (University of Toronto)

Academic institution under whose auspices the research will be conducted:

1. College of Medicine, University of Malawi
   1 Mahatma Ghandi Road
   P Bag 360, Chichiri, Blantyre 3

Collaborating Institutions

1. Liverpool School of Tropical Medicine, United Kingdom.
2. University of Liverpool, United Kingdom
3. University of Toronto, Canada

Signature: Mwayiwawo Madanitsa

Confidentiality statement: This document contains confidential information that must not be disclosed to anyone other than the grant sponsor, the investigational team, Malawi CoM research support office, regulatory authorities, and members of the Research Ethics Committees.

Table of Contents

1. Study Synopsis 5
2. Executive Summary 6

Diagram of main study visits 8

Summary of scheduled study visits and procedures 9

3. List of abbreviations 10
4. Background 11

Malaria in Pregnancy 11

Current challenges with IPTp-SP and alternative strategies 11

Impact of intrauterine insults on long-term neurodevelopment 12

Effect of Pregnancy Associated Malaria and Repeated Antimalarial Treatment on Infant Neurocognitive Development 13

Effect of Pregnancy Associated Malaria on Infant Susceptibility to Malaria 14

Effect of Clinical Malaria on Early Childhood Neurocognitive Development 16

5. Rationale and hypothesis 16
6. Objectives 18

Primary Objectives 18

Secondary Objectives 18

7. Overview Design 18
8. Methods Parent trail (ISTp Study). 19

Brief synopsis parent trial 19

Objectives and endpoints parent trial 20

Primary objective 20

Primary endpoints 20

Secondary objectives and endpoints 20

Study Population parent trial 20

Study arms parent trial 21

Concept of ISTp parent trial 21

Schedule parent trial 21

Study sites parent trial 21
Randomization parent trial 21
Procedures parent trial 22
9. Methods Birth Cohort 22
Study location birth cohort 22
Study population birth cohort 23
Inclusion criteria 23
Exclusion criteria 23
Procedures Birth Cohort 23
Consent procedures 23
Follow-up schedule and visits 23
Compliance with follow-up schedule birth Cohort 25
10. Study period and Timeline 25
11. Sample size 26
Primary objectives: Neurocognitive evaluation 26
Secondary objectives: Early childhood malaria susceptibility 26
12. Data collection 28
Epidemiological measures 28
In-utero exposures 28
Infant outcome measures and determinants 28
13. Data management 28
Managing and preserving data 28
Data management 28
Archiving 29
14. Data analysis 29
Statistical support 29
Analytical approach 29
Adjustment for confounding 29
Planned subgroup analyses 30

Statistical methods 30
15. Result presentation and dissemination 30
16. Ethical considerations..... 31
Ethical review and research governance arrangements 31
Risks and benefits 31
Informed consent..... 31
Confidentiality..... 32
Knowledge of mother and infant HIV status 32
Compliance with guidelines 32
17. Possible constraints..... 32
18. Management of the Project..... 32
Roles investigators and senior support staff 32
Legal Sponsorship..... 33
Administration..... 33
19. Budgetary estimates..... 33
20. Justification of the budget 34
Personnel 34
Equipment and services 34
Clinical Supplies 34
Participant reimbursements 34
Office consumables 34
21. References..... 35
22. APPENDIX I (English Information Sheet) 38
23. APPENDIX II (English Consent Form)..... 40
24. APPENDIX III (Chichewa Information Sheet) 41
25. APPENDIX IV (Chichewa Consent Form)..... 44

1. Study Synopsis

**Study Title**	The Effect of Pregnancy Associated Malaria on Early Childhood Neurocognitive Development: an Observational Birth Cohort Study
**Acronym**	PAMaNeD
**Protocol ref. no.**	
**Study Design**	Observational Prospective Birth Cohort
**Planned Sample Size**	500
**Follow-up duration**	From twelve months to 24 months of age
**Planned Study Period**	18 months

2. Executive Summary

Introduction: Malaria is known to contribute substantially to the high mortality in children under-five years of age. Newborns are protected from severe malaria during the first few months of life through the transfer of maternal antibodies and the presence of relatively high concentrations of foetal haemoglobin which makes the red cell less susceptible to *Plasmodium falciparum*. When these protective factors have waned by 4 to 6 months, this is followed by a vulnerable period of increased susceptibility before the child has had the opportunity to acquire protective immunity resulting in a high burden of severe malaria in older infants.

Animal model studies suggest that offspring born from pregnancies with placental malaria have impaired neurocognitive development. We hypothesize that a infants born to mothers infected with malaria during pregnancy may suffer from similar neurocognitive insults. In addition to the timing of infection, the frequency and severity of maternal infection may also be important determinants of the degree of insult. For example it is unknown if low-density asymptomatic PCR positive sub-microscopic infections in the mother may result in significant restriction in neurocognitive development. If it does, prevention of sub-microscopic infection in pregnancy would be important.

In addition to the risk of impaired neurocognitive development, studies suggest that infants born from pregnancies with placental malaria have a higher probability of developing clinical malaria and malaria associated anaemia than those born from pregnancies without placental malaria. A growing body of literature also suggests that cognitive abilities are adversely affected following cerebral malaria or uncomplicated malaria infection which persists even after successful treatment and recovery from infection. These two suggestions propose a potentially vicious ex-utero pathway of continuing neurocognitive insult following in-utero malaria exposure.

These findings could have important implications for the design the control strategies for the prevention of malaria in pregnancy and the prevention of malaria and fostering of neurocognitive development in early childhood.

Methods: We will follow 500 infants born to women enrolled in a large randomised controlled trial comparing the impact of the standard strategy of Intermittent Preventive Treatment with Sulphadoxine-Pyrimethamine in pregnancy (IPTp-SP) to a novel strategy of intermittent screening and treatment in pregnancy (ISTp). Gestational age will be determined by ultrasound and each woman will be seen at least 3 times during pregnancy and again delivery. Detailed information on infection incidence during pregnancy determined by detection PCR, malaria smear, rapid diagnostic tests, and at delivery by placental histology will be available. All infants will be followed for one year and seen at 12, 18 and 24 months of age. Passive case detection for illnesses will be used in between scheduled follow-up visits.

Primary Objectives

1. To determine if neurocognitive development in early childhood is affected, independent of low birth weight, by pregnancy associated malaria
2. To determine if the impact of pregnancy associated malaria on neurocognitive development, independent of low birth weight, is affected by repeated exposure to antimalarial drugs taken by the mother during the 2nd and 3rd trimester.

Secondary Objectives

1. To determine the effects of maternal malaria infection, during the 2nd and 3rd trimester and at delivery, on the risk and frequency of clinical malaria, anaemia (Hb< 8g/dL) and growth during early childhood.
2. To determine if early childhood neurocognitive development is affected by clinical malaria and anaemia during early childhood.

International collaboration

The study is part of an ongoing collaboration between Dr Victor Mwapasa from the College of Medicine, the National Malaria Control Programme of the Ministry of Health, the Liverpool School of Tropical Medicine (LSTM), the University of Liverpool and the University of Toronto.

Funding: The parent clinical trial is supported by EDCTP and the Malaria in Pregnancy Consortium (LSTM). Funding for the birth cohort ($ 250,000.00) is requested from Grand Challenges Canada, Saving Brains.

Diagram of main study visits

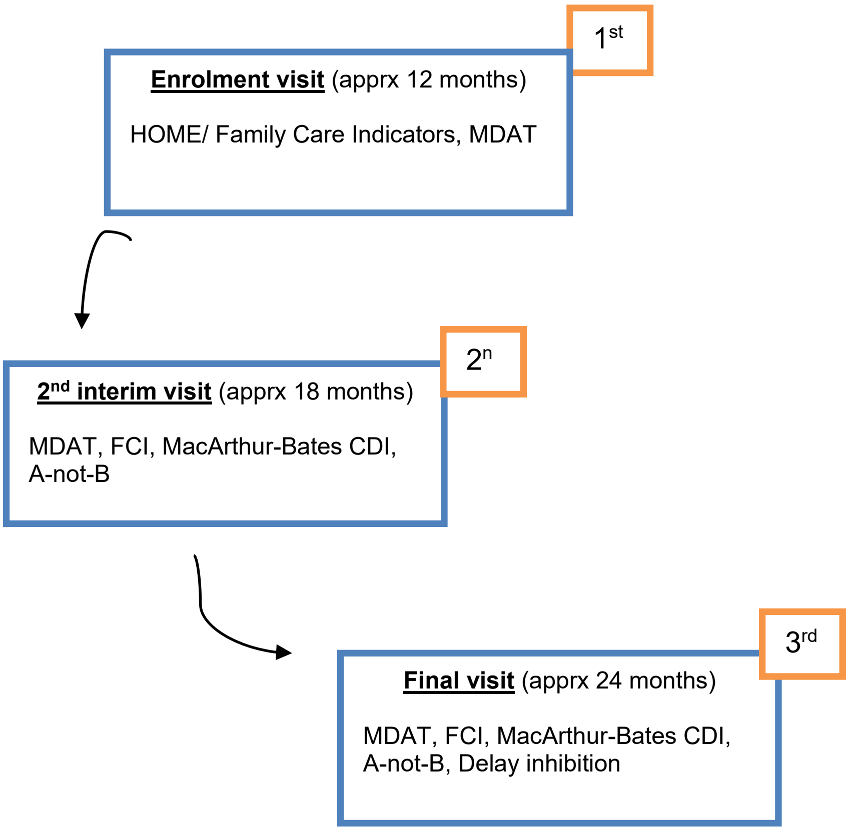

Summary of scheduled study visits and procedures

	Parent trial				Birth Cohort Follow-up		
	**Mother Booking visit** (16 to 28 weeks)	**Enrolment visit** (Delivery)	**7-day postnatal visit** (7 days)	**6-week postnatal visit** (6 -8 weeks)	**1st interim visit** (12 months)	**2nd interim visit** (18 months)	**Final visit** (24 months)
**Actions**							
Consent	X			X			
Eligibility confirmed		X					
**Measures**							
Maternal malaria blood samples (RDT, smear, PCR)		X					
Placental malaria sampling (histopathology)		X					
Birth weight		X					
Gestational age		X					
Congenital anomalies		X	X	X			
Neonatal jaundice		X	X				
Clinical malaria and other childhood illnesses assessment					X	X	X
Growth and nutrition assessment (weight, height, MUAC)		X			X	X	X
**Neurocognitive Development**							
HOME/Family Care Indicators					X	X	X
MDAT					X	X	X
McArthur-Bates, A-not-B, Delay inhibition						X	X

3. List of abbreviations

ACTs	Artemisinin-based Combination Therapies
IPTp	Intermittent Preventive Treatment in pregnancy
IPTp-SP	Intermittent Preventive Treatment in pregnancy with Sulphadoxine-Pyrimethamine
ISTp	Intermittent Screening and Treatment in pregnancy
MDAT	Malawi Developmental Assessment Tool
SP	Sulphadoxine-Pyrimethamine

4. Background

Malaria in Pregnancy

Worldwide about 515 million episodes of clinical *P. falciparum* malaria occur each year and 800,000 to 2 million people die from the disease. Most cases and fatalities occur in Sub-Saharan Africa, 90% of whom are children under five years of age.[1]

Although, adolescents and adults living in these areas rarely develop clinical malaria when infected, during pregnancy, women are highly vulnerable to the adverse consequences of infection especially during the first pregnancy.[2, 3] An estimated 32 million pregnant women are at risk of *P. falciparum* malaria infection, resulting in 23 million live births every year in Sub-Saharan Africa.[4] In antenatal clinics in southern Malawi in 2010, approximately one in five women had evidence of malaria infection in the 2nd trimester and more than a third of the primi,- and secundigravidae had evidence of active or recent infections at delivery (Kalilani & ter Kuile, personal communications).

P. falciparum infection during pregnancy is characterized by the sequestration of antigenically unique *plasmodium*-infected erythrocytes (IEs) in the placental intervillous spaces[5] mediated by adhesive interactions between parasites antigens on the surface of erythrocytes, *P. falciparum* erythrocyte membrane protein 1 (PfEMP1) and a glycosaminoglycan receptor expressed by syncytiotrophoblasts lining the placental intervillous spaces called chondroitin sulphate A (CSA).[2, 6] This feature makes placental malaria different from the malaria that commonly infects under-five children or adults.[6, 7]

The well-recognised consequences of *P. falciparum* infection during pregnancy include maternal anaemia, premature birth and foetal growth restriction resulting in low birth weight and higher perinatal morbidity and mortality.[3, 8, 9] These effects are most severe in first and second pregnancies.[7] Additional consequence of placental malaria that are now receiving much more attention are the effect of *in utero* exposure to malaria on neurocognitive development [10, 11] and the potential long-term effect on susceptibility to malaria infection in early childhood. [12, 13, 14]

Current challenges with IPTp-SP and alternative strategies

Despite the high resistance patterns to SP and the consequent compromised efficacy of SP in the case management of symptomatic malaria in children, IPTp-SP has previously remained effective in most parts of Africa. This is probably due to the fact that pregnant

women have more immunity and lower parasite densities than young children.[15] However, current evidence suggests that the effectiveness of IPTp-SP in eastern and southern Africa is waning. A recent analysis of the effectiveness of IPTp-SP between 1996 and 2006 in Malawi, showed that the impact of IPTp reduced dramatically since 2002, and has reached levels where it is no longer protective.[16] These findings in Malawi are of grave concern and are consistent with recent observations from northern Tanzania.[17] Preliminary results of a currently on going observational study assessing the efficacy of IPTp-SP in HIV seronegative women has shown the prevalence of placental malaria standing at 1 in 3 women (Linda Kalilani, personal communication), further emphasising the desperate situation malaria control in pregnancy faces, and the need for an alternative strategy.

In areas with high SP resistance where IPTp-SP is failing, the alternative options are limited to either replacing SP with other drugs for IPTp, or considering alternative strategies to replace IPTp. In addition, it has been noted that the transmission of malaria is declining in many parts of Africa, including southern Malawi, and is likely to decline further with the provision of funds for malaria control and elimination initiatives.[16] This will also result in reductions in the number of women at risk for malaria infection during pregnancy, reducing the potential impact and cost-effectiveness of presumptive approaches such as IPTp.

There is increasing interest in using screening approaches for the control of malaria in pregnancy.[18, 19] One strategy that has been proposed is Intermittent screening and treatment in pregnancy (ISTp) which involves screening for malaria as part of focused antenatal care using appropriate diagnostics and treating parasiteamic women with long acting Artemisinin-based Combination Therapies (ACTs) to clear the existing infections, while providing additional post-treatment prophylaxis for 3 to 6 weeks. The screening ensures that only parasiteamic women receive treatment, whereas women without evidence of malaria i.e. lower risk groups such as the multigravidae or women protected by ITNs are not unnecessarily exposed to antimalarial drugs. Evidence from a non-inferiority trial between ISTp and IPTp conducted in Ghana involving 3,333 women, suggests that ISTp may be an effective strategy for some parts of Africa.[18, 19]

Impact of intrauterine insults on long-term neurodevelopment

Infections in pregnancy have long been recognized as important determinants of maternal and fetal morbidity and mortality, particularly in the neonatal period. However, the impact of infection and inflammation on long-term neurodevelopment is less well understood. A recent systematic review examining the long-term consequences of intrauterine and neonatal insults looked at the risk and severity of sequelae following neonatal insults (e.g. sepsis,

tetanus, meningitis, jaundice, preterm birth), congenital infections (cytomegalovirus, toxoplasma, syphilis, rubella) and HIV[37]. After including 22,161 survivors of intrauterine or neonatal insults, the pooled risk estimated of at least one sequela associated with one or more insults (excluding HIV) was 37% (95% CI, 27-48%). The risk was not significantly affected by region, duration of follow-up, study design, or period of data collection. 59% of sequelae were learning difficulties or cognitive or developmental delay. These data demonstrate that intrauterine or neonatal insults can have subtle impacts on development that may not be appreciated using existing outcome measures. Importantly, no human data is available on whether exposure to malaria in pregnancy will affect fetal brain development. Recent data from a murine model of placental malaria indicate that malaria exposure in utero results in persistent deficits in memory and affective-like behaviour compared to uninfected controls (Kain, personal communication). This effect was mediated through activation of the complement system, an important component of the innate immune response to infection.

Effect of Pregnancy Associated Malaria and Repeated Antimalarial Treatment on Infant Neurocognitive Development

The consequent uterine placental insufficiency due to maternal malaria leading to adverse fetal outcomes is well recognized in literature, in particular fetal growth restriction and preterm delivery. In malaria non-endemic populations, utero-placental insufficiency has been associated with impaired neurocognitive development. [43] As such, low birth weight infants (< 2500g) have been demonstrated to suffer neurocognitive developmental impairment stemming from in-utero trophic insults.[44] However, it is postulated that fetal inflammatory responses in response to local placental pro-inflammatory cytokine changes during infections in pregnancy, may induce intrauterine neuronal damage with consequent adverse neurocognitive outcomes [45] Stimulation of the pro-inflammatory cytokine cascade due to exposure to malaria antigens directly (in congenital malaria though rare) or indirectly (in placental and maternal peripheral malaria) as well as placental inflammatory cytokines produced in pregnancy associated malaria, may have a potentiating effect to adverse neurocognitive outcomes in infants in pregnancy associated malaria other than low birth weight. To this extent, it is estimated that infants born to primigravid and secundigravida mothers will be at greater risk of neurocognitive impairment through this pathway as such mothers are more susceptible to malaria infection. Antimalarial therapies in chemoprophylaxic and treatment programmes in pregnancy have been widely studied to determine the safety and efficacy of antimalarials in pregnancy, reduction of maternal malaria associated morbidity and mortality and adverse birth outcomes. However, there is

little evidence that has evaluated the impact of prolonged in-utero exposure to antimalarials on neurocognitive development.

Effect of Pregnancy Associated Malaria on Infant Susceptibility to Malaria

There is mounting, albeit inconclusive, evidence that maternal malaria infection increases infants' susceptibility to malaria infection through the pre-natal exposure to malaria antigens and possible immune-sensitisation. An early study conducted in Cameroon found no significant difference in the frequency of malaria between infants born to mothers with placental malaria and infants born to mothers without placental malaria during the first twenty four months of life.[20] However, the age-specific prevalence of *P. falciparum* malaria parasitemia was consistently higher between 4 and 18 months of age among infants born to mothers with placental malaria. The overall malaria-free survival rates were not significantly different between the two groups of infants, although a considerable difference was observed between 5 and 8 months of age among infants born to placental malaria-infected mothers.[20]

In a subset analysis of the same infant cohort, cord serum reactivity against specific parasite isolates (CSA binding-parasites) was related to a younger age at first parasitemia in the infant and associated with increased frequency of parasitemia during infancy.[21] The investigators inferred that placental malaria occurring during the last months of pregnancy may have increased anti-CSA-binding IgG antibodies which are transferred to the infant but may not be effective against other antigenically distinct parasites (non CSA binding parasites) which are known to cause malaria in children. The investigators also suggested that increased infant susceptibility to malaria may be due to immunologic tolerance or congenital transmission of parasites.[21] However, the study did not conduct any immunological studies in infants to support their suggestions.

A subsequent study conducted in Muheza District in Tanzania, an area with intense malaria transmission, with an entomological inoculation rate of 400 infective mosquito bites each year, found that infants born to mothers who had evidence of placental malaria at delivery were 41% more likely to experience their first parasitemia at a younger age compared to those who were born from mothers who did not have placental malaria at delivery.[22] However, the increased risk was confined to infants born to multigravid women and not primi and secundigravid women. This finding was surprising considering the known high risk of malaria in primigravidae and high incidence of post-neonatal infant mortality of infants born to malaria infected primigravid mothers with malaria.[23, 24, 25] Nevertheless, the study findings

seems to support the earlier hypothesis that high levels of anti-CSA-binding IgG antibodies, which presumably are more prevalent in multigravid women with malaria and which are subsequently transferred to the infant, may not be effective in providing protective immunity to the infants against antigenically distinct parasites.

A recent study conducted in Kenya has shown that a subset of children exposed to malaria in-utero acquire a tolerant phenotype to blood-stage antigens that persists into childhood and is associated with an increased susceptibility of the child to malaria infection and anemia.[26]

Importantly, these effects are independent of low birth weight; i.e. they also occur in term children with normal birth weight.

Two more recent studies have provided further evidence suggesting that malaria in pregnancy increases infant susceptibility to malaria infection. A Gabonese study found that offspring of mothers with placental *P. falciparum* malaria infection were about twice more likely to have clinical malaria in the first 30 months of life than those born to uninfected women. In addition, the median parasite density was more than three times higher in infants born to malaria-infected mothers.[27, 28]

Similar to the Tanzania study,[22] the association between placental malaria and subsequent infant malaria infection was stronger among multigravid than primigravid women[27].

A previously study conducted to determine the differences in the humoral immune responses to 7 different Plasmodium *falciparum* epitopes between infants born to mothers who had placenta malaria and those who did not have placenta malaria found that placental malaria was associated with diminished antibody levels to all of the epitopes tested, especially with infants aged >4 to 12 months and the difference was statistically significant for four of the seven epitopes. [29] These findings suggest that placental malaria can negatively influence the development of the humoral immune responses to malaria in infants, especially those with potential vaccine candidacy.

One of the most detailed and comprehensive studies conducted on this topic to date, the results of which have just been published, has shown that depending on the nature of *in utero* exposure to malaria antigen, the foetal adaptive cell-mediated immunity could result in either only being exposed without being sensitised or being exposed and sensitised. The study showed that the exposed but not sensitized neonates had T cells which had developed some anergy but had an increased ability to produce IL-10 which they suggested to be an indication of immune tolerance. The investigators also showed that this exposed but

not sensitised group was more susceptible to malaria and anaemia than the other two groups suggesting that the tolerance they may have developed due to *in utero* exposure to malaria antigen thus affecting how their immune system responds to subsequent malaria infections.[26]

This evidence suggests that the successful prevention or in part, the reduction, of intra-uterine immunomodulation due to exposure to malaria antigens, through at least the second and third trimesters of pregnancy, could potentially reduce the susceptibility of malaria and anaemia in infancy and later childhood especially under the age of five years.

Effect of Clinical Malaria on Early Childhood Neurocognitive Development

The adverse sequelae of severe malaria on neurocognitive function are well documented. An early systematic review concluded that severe malaria disease has continuing adverse effects on cognition[34]. A more recent systematic review by Fernando et al in 2010 showed evidence that cognitive abilities and performance were adversely affected in both severe and uncomplicated malaria with persistent impairment of cognitive ability and school performance even after recovery [35].

The majority of literature on neurocognitive effects of malaria infection has been conducted in children of school going age with a focus on severe malaria disease. Very few studies have been conducted to evaluate the effect of malaria exposure on neurocognitive development in early childhood. One of the most recent studies to date conducted in Zambia showed an association between early childhood exposure to malaria and some domains of pre-school development[36]. However, a direct causal relationship could not be inferred in the study as malaria exposure in the empirical analysis did not measure the individual exposure of each child but rather the cluster level exposure.

5. Rationale and hypothesis

The previous prenatal studies above suggest that prenatal exposure to maternal malaria events may affect early childhood neurocognitive development independent of low birth weight with a likely causal relationship. However, most of these studies were restricted to the assessment of maternal exposure to malaria at birth only; i.e. a single time point to measure exposure. The timing of infection during pregnancy and the relative age of the developing fetus could be important determinants of the relationship. In addition to the timing of

infection, the frequency and severity (e.g. low vs high density infections) of maternal infection may also be important determinants of the degree of neurocognitive insult inflicted in utero. Previous studies have not assessed if low-density asymptomatic PCR positive sub-microscopic infections in the mother have an effect on in utero neurocognitive development with possible continued effects into early childhood. A common feature of all previous studies is the use of relatively insensitive measures to diagnose malaria infection in pregnancy (placental or peripheral blood smear).

The neurocognitive impact of pregnancy associated malaria beyond low birth weight as well as uncomplicated malaria disease in early childhood has not been fully investigated. If a casual relationship is identified, the impact of pregnancy associated malaria, independent of low birth weight, would have great ramifications on loss of cognitive potential and subsequent aggregate cognitive capital in malaria endemic populations with consequent reduction of microeconomic returns on education and macroeconomic performance of such populations. This effect would be further compounded if a comprehensive relationship between early childhood exposure to malaria and impaired neurocognitive development is established.

This birth cohort is likely to provide an important contribution to highlighting the unconventional burdens of malaria in early childhood and shed further light on the main risk factors for neurocognitive development restriction in early childhood by determining the relative contribution of congenital infections, acquired infections after birth and of the role of pre-natal antimalarial drug exposure. We propose to address these questions by using antenatal in-utero malaria exposure repeat sampling for malaria by microscopy and RDT, but also by the more sensitive PCR methods and placental histology, thus minimizing misclassification bias and by combining epidemiological findings with neurocognitive studies (thus improving biological plausibility of our hypothesis). Longer term assessment of benefits and safety of ISTp with DHA-piperaquine

The infant and maternal follow-up in the main pregnancy study stops at 6 weeks post-partum. Thus the current proposed study adds valuable infant follow-up time allowing for more in-depth assessment of the risk and benefits of Intermittent Screening and Treatment in pregnancy with DHA-piperaquine (a relatively new antimalarial in Africa) on infants' neurocognitive development and susceptibility to malaria infection.

6. Objectives

Primary Objectives

1. To determine if neurocognitive development in early childhood is affected, independent of low birth weight, by pregnancy associated malaria
2. To determine if the impact of pregnancy associated malaria on neurocognitive development, independent of low birth weight, is affected by repeated exposure to antimalarial drugs taken by the mother during the 2nd and 3rd trimester.

Secondary Objectives

1. To determine the effects of maternal malaria infection, during the 2nd and 3rd trimester and at delivery, on the risk and frequency of clinical malaria, anaemia (Hb< 8g/dL) and growth during early childhood.
2. To determine if early childhood neurocognitive development is affected by clinical malaria and anaemia during early childhood.

7. Overview Design

This is an observational prospective birth cohort study in which newborns will be recruited at 12 months from a larger clinical trial that compares IPTp-SP with intermittent screening and treatment approaches (ISTp) for the control of malaria in pregnancy funded by EDCTP ('ISTp' Study; NHSRC number 916). This 'parent trial' involves regular follow-up of the pregnant women during pregnancy (3 to 4 times) and again at delivery. During these scheduled visits blood samples are collected for malaria smears and detection PCR (and RDTs as point of care in the ISTp arm). The smears and PCR are not point-of-care and are read after delivery. At delivery malaria infection is detected by peripheral smear, placental smears, RDTs, placental histology and PCR. The women will also be seen at any point during follow up if they have symptoms of malaria and blood samples will also be collected for malaria smears and detection PCR.

Because of the special focus on malaria diagnostics requiring frequent measures for malaria infection during pregnancy, all the infants enrolled in the birth cohort are born to pregnancies that have well documented malaria exposure histories during pregnancy and at delivery. Malaria diagnosis will be performed using RDTs, PCR, placental histopathology and conventional microscopy. In addition, detailed clinical and demographic data will be collected

including maternal gravidity, age, HIV and syphilis status, malaria exposure, use of anti-malarial interventions such as bed nets and intermittent preventive therapy, maternal socio-economic status, and maternal education.

Below we first describe the details of the ISTp study in pregnant women and then continue describing the details of the birth cohort.

8. Methods Parent trail (ISTp Study).

Brief synopsis parent trial

An open-label two-arm parallel-group multicentre individually randomised controlled superiority trial conducted in two rural sites in Blantyre district and one site in Chikwawa district, southern Malawi with high levels of resistance to SP and moderate levels of *P.falciparum* malaria transmission. The trial compares the efficacy, safety and cost-effectiveness of the current policy of IPTp-SP with a new strategy consisting of intermittent screening and treatment in pregnancy with rapid diagnostic tests and treatment with RDT positive women with DHA-piperaquine (DP) (ISTp-DP). Women will be seen at least 3 times during pregnancy, and at birth. Women attending early in pregnancy (<=24 weeks gestation) will be seen at least 4 times during pregnancy. Infants will be followed for 6 weeks. The study population includes HIV-negative women, gestational age 16-28 weeks inclusive (based on dating ultrasound), who are scheduled to receive their first course of SP during this pregnancy. Recruitment will be stratified by gravidity groups: primi- and secundigravidae (G1+2), and multigravidae (G3+).

Among G1+2, the study is designed to detect 25% reduction (from 40.3% to 30.2%) in the risk of preterm birth or fetal growth restriction, defined as a composite endpoint of small-for gestational age, low-birth weight or preterm births (90% power). Among multigravidae the study is designed to detect at least a 50% reduction in active or recent infection assessed at delivery by placental histopathology, blood smear, PCR or RDT. The planned sample size is 1,665, including 1155 G1+2 and 500 G3+. The sample size estimates are based on current year-round observations in the same study sites. The project also includes a feasibility and a cost-effectiveness component.

Objectives and endpoints parent trial

Primary objective

To compare the efficacy of scheduled intermittent screening with malaria rapid diagnostic tests (RDTs) and treatment of RDT-positive women with dihydroartemisinin-piperaquine (ISTp-DP) with intermittent preventive treatment with sulphadoxine-pyrimethamine (IPTp-SP) in the second and third trimesters on adverse birth outcome and malaria infection at term among HIV-negative women protected by insecticide–treated bed nets.

Primary endpoints

First and second pregnancies: adverse birth outcome (composite of small-for-gestational age (SGA), low birth weight or preterm birth); *Multigravidae (G3+):* Active or recent infection assessed at delivery by placental histopathology or RDT.

Secondary objectives and endpoints

To determine if ISTp-DP has greater efficacy than IPTp-SP in terms of placental malaria (in G1 and G2), maternal malaria infection at delivery, mean birth weight, low birth weight (<2,500 grams), gestational age, mean gestational age at birth, pre-term birth (<37 weeks), small for gestational age, mean maternal haemoglobin at birth; anaemia (Hb ≤ 11 g/dL) at birth, moderate to severe anaemia (Hb ≤ 8g/dL); miscarriage, stillbirths; neonatal deaths; clinical malaria episodes during the second and third trimesters of pregnancy; third trimester mean maternal haemoglobin, anaemia (Hb ≤ 11 g/dL) and moderate to severe anaemia (Hb ≤ 8g/dL) and a composite endpoint of the primary endpoint plus fetal loss; severe cutaneous skin reaction in the mothers; other serious adverse events in the mothers; minor adverse events in the mothers by day three after study drugs given; congenital malformation at birth and by day 28; neonatal jaundice at day one or day seven; incidence of anaemia, and clinical malaria in babies up to the age of eight weeks.

Study Population parent trial

HIV-negative women, gestational age 16-28 weeks inclusive, who are scheduled to receive their first course of SP during this pregnancy and who agree to deliver in the hospital or study clinics. Dating ultrasound scans will be performed to assess gestational age.

Study arms parent trial

1. IPTp-SP: 3 to 4 doses of IPTp with SP as part of FANC (according to current practice in Malawi women coming early get 4 doses, women coming from 24 weeks get 3 doses of SP)
2. ISTp-DP: 3 or 4 scheduled screenings visits with an RDT and treatment with DHA-PQ if they are RDT-positive.

Concept of ISTp parent trial

The concept of ISTp is to provide screening as part of focused antenatal care using appropriate diagnostics to detect malaria in pregnant women, and treating parasitaemic women with long acting artemisinin-based combination therapies (ACTs) to clear the existing infections, while providing additional post-treatment prophylaxis for 3 to 6 weeks.

Schedule parent trial

- Three or 4 scheduled visits spread over the 2nd and 3rd trimesters approximately 4 to 8 weeks apart mirroring the appointment schedule for FANC in Malawi. Women attending early in the second trimester (16-23 weeks) will make 4 scheduled visits.
- Women will be encouraged to attend the study clinic for assessment if they feel unwell during the study period. Participants who deliver at home will receive a home visit within 24 to 72 hours. Participants receiving DHA-PQ will also receive a home visit on day two to encourage and assess drug adherence and assess tolerability.
- Newborns will be seen at approximately seven days and six weeks after delivery.

Study sites parent trial

Antenatal clinic sites at Mpemba, Madziabango and Chikhwawa District Hospital are involved.

Randomization parent trial

Participants will be randomly allocated by two randomization sequences which will be computer-generated by the study statistician at Liverpool School of Tropical Medicine, one for women in their first and second pregnancies and another for multigravidae. Stratified block randomization (varying block sizes) will be used (by study site). Recruitment will be ‘competitive’ between study sites. The study uses opaque envelopes, numbered

sequentially, with the allocated group code. For each newly enrolled participant, an envelope from the correct sequence (according to gravidity group) is opened sequentially to identify the group that they are allocated to, thus concealing the upcoming allocation from the participants, clinic staff and study staff. The number on the envelope represents the study number allocated to that participant and acts as the unique identifier in the trial.

Procedures parent trial

All women will be screened for malaria at least 3 to 4 times during the 2nd and 3rd trimester for malaria parasitemia. On delivery, the placenta will be collected for histopathological analysis to establish evidence of placental malaria and a thick blood film, rapid malaria diagnostic test (RDT) and polymerase chain reaction (PCR) from the mother to test for evidence of malaria infection (active or past) at delivery. Participants who deliver at home will receive a home visit within 24 hours (maximum time a week).

In the ISTp arm the screening is part of point-of-care (as RDTs will be used). Women in the IPTp arm will follow the same schedule and have blood taken for later microscopy and PCR, but no RDTs will be done (i.e. not used for point of care), unless the women has fever or a history of fever.

Participants will receive all usual antenatal care according to local policy, including standard clinical examinations, tests and necessary treatment. Participants receiving DHA-PQ will also receive a home visit on day two after the first dose of treatment to encourage and assess their adherence to the study medication, and assess tolerability.

Haemoglobin levels will be tested at enrolment and in the third trimester, and those found to be anaemic will be treated with haematinics according to Government guidelines.

9. Methods Birth Cohort

Study location birth cohort

The birth cohort study will be conducted in two of the health facilities; namely Mpemba and Chikwawa District Hospital that collectively cater predominantly for rural and periurban populace.

Study population birth cohort

The participants of this study are infants born to mothers participating in the ISTp study, NHSRC number916.

Inclusion criteria

1. Ability of the mother to provide written informed consent
2. Resident within the catchment area of the clinical study sites and will be resident for the following 12 months from enrolment
3. Mother/caretaker willing to adhere to the study requirements
4. Previously enrolled in the MIPc RCTs of ISTp vs IPTp

Exclusion criteria

i. Any major congenital abnormalities

Procedures Birth Cohort

Consent procedures

Infants shall be recruited at 12 and 18 months of age, as the initial study visit. Informed consent will have been obtained from the primary care giver.

Follow-up schedule and visits

Visit 1: Enrolment (12 months)

Mothers, who were previous participants in the parent trial, will be approached for inclusion of their child in the study. A detailed history of any illnesses the child has suffered from the last visit in the main trial (i.e. 6 week postnatal visit) to date will be recorded and corroborated with documentation in the child's health passport book. All medications that the child has received will also be documented.

Procedures:

The Malawi Developmental Assessment tool will be used to assess development at 6 monthly intervals. This has been validated and used in numerous studies in Malawi. It takes 30 mins to conduct and has some tools which are required which are all local to Malawi and contained in a basket which the assessor uses during the assessment.[30]

The Home Observation for Measurement of the Environment (HOME) [31] is a 58-question assessment of the stimulation and learning opportunities offered by the child's home environment. It has been used in many different countries in the world and has been adapted and validated for use in Malawi as part of the ILINs trial in Mangochi. Family Care Indicators (FCI) will also be used and may supplement or replace HOME in the event a visit to the child's home is not possible. Like the HOME, the FCI assesses the ability of the household to meet the physical, mental and social needs of the child but the questionnaire can be completed without naturalistic observation of the child in the his or her home. The FCI have also been adapted and validated for use in Malawi as part of the ILINs trial.

Subsequent and final visits 2 and 3:

Babies will be followed-up at 18 and 24 months. Any infant deaths occurring before the visit will be recorded, having been established through report by the care giver or upon community follow-up for a missed visit; the date and probable cause of death will be ascertained by verbal autopsy.

Care givers will be asked about any symptoms or illnesses participants have had since the first visit of the study, and any medications administered which were either prescribed or given at home without prescription as well as use of traditional remedies. Treatment of any illness identified at this visit will be provided per national policy guidelines.

Procedures:

Assessment of neurocognitive development using the Malawi Developmental Assessment Tool will be conducted at each interim EPI visit to evaluate age specific neurocognitive development. FCI will also be conducted at each scheduled visit to capture any changes that may occur in the child's environment.

The MacArthur Bates Communication Developmental Inventory[32] has been used in the ILiNs trial in Malawi to assess language development in children age 18 months in Malawi. The interview consists of 100 vocabulary items, 6 gesture items, and 5 grammatical items. Scored by number obtained correct (will be used at 18 months and 24 months)

The A not B task is a simple task where an object is hidden first in one location (A) and then in another location (B). Infants who continuously search at location A may have poor motor inhibition demonstrating difficulties with executive function. This test has been demonstrated to be sensitive for even very young infants. Competence in the task has been shown by 12

months. Scored best of six. (used at 18 months and 24 months). This has been used in the ILiNs study in Malawi and therefore is already adapted and validated.

Delayed inhibition task: Snack delay task – the tester places a snack under a clear cup (or in the child's hand) and tells the child to wait with delays of 5, 15, 30 and 45 s (used at 24 mo). This has been validated in Uganda and will be validated for use in Malawi.

Unscheduled visits

Mothers will be encouraged to bring any sick infants to the study clinics in between the scheduled visits for clinical management. At each unscheduled clinic visit a heel prick sample will be taken for malaria RDT and estimation of hemoglobin. Appropriate management shall be instituted and details of the event will be recorded on dedicated study forms.

Compliance with follow-up schedule birth Cohort

The visits will be planned to coincide with the children's scheduled EPI visits. Infants who miss a scheduled visit will be visited at home if so required. All Transport costs at visits will be reimbursed. Two recently completed large cohort studies in these areas, each of which achieved high rates of follow-up (94%+ by 6 months and 89% by 12 months) (IPTpd study; Kamija Phiri and ter Kuile, personal communications; and the SevAna study, thus an estimated 80% retention is a conservative estimate.

10. Study period and Timeline

The project is expected to run for 22 months, with the initial 12 months dedicated to recruiting infants, the follow-up of these infant for 12 months and the completion of follow-up and termination of the study in the last 6 months. The remaining 4 months will be focused on finalising the analysis, write-up and presentation of the study findings.

11. Sample size

Primary objectives: Neurocognitive evaluation

A sample size of 400 (100 infants born to mothers infected with malaria, 300 infants born to mothers uninfected with malaria) will have 80% power to detect a difference of 0.8 in mean test score in the A-not-B task assuming a standard deviation of 2.36 in each group.

Secondary objectives: Early childhood malaria susceptibility

Assuming that infants potentially exposed to placental malaria have a twofold risk of malaria[27] compared to unexposed infants, there is more than 80% power to detect a twofold risk of clinical malaria in the group potentially exposed. Using STATA/IC 12.1:

```
. sampsi 0.2 0.4, alpha(.05) n1(300) n2(100)

Estimated power for two-sample comparison of proportions

Test Ho: p1 = p2, where p1 is the proportion in population 1
                    and p2 is the proportion in population 2
Assumptions:

         alpha =   0.0500  (two-sided)
            p1 =   0.2000
            p2 =   0.4000
sample size n1 =      300
            n2 =      100
         n2/n1 =     0.33

Estimated power:

         power =   0.9608
```

Enrolment procedures: Statistical considerations

403 infants are potentially available for enrolment at 12 months. However, given an estimated successful re-recruitment rate of 58% based on previous enrolment estimates

from the parent trial and additional attrition rate of 20% through the study, the sample size at the end of the study will have inadequate power to address the primary research question:

```
. sampsi 0.2 0.4, alpha(.05) n1(124) n2(62)

Estimated power for two-sample comparison of proportions

Test Ho: p1 = p2, where p1 is the proportion in population 1
                    and p2 is the proportion in population 2
Assumptions:

         alpha =   0.0500  (two-sided)
            p1 =   0.2000
            p2 =   0.4000
sample size n1 =      124
            n2 =       62
         n2/n1 =     0.50

Estimated power:

         power =   0.7700
```

As such, additional infants shall be enrolled into the study at 18 months thus allowing for an adequate sample size with sufficient power to answer the primary objectives as stated above. There will still be adequate power to compare primary outcomes at 12 months. Assuming 58% re-recruitment for 12 months:

```
. sampsi 0.2 0.4, alpha(.05) n1(155) n2(78)

Estimated power for two-sample comparison of proportions

Test Ho: p1 = p2, where p1 is the proportion in population 1
                    and p2 is the proportion in population 2
Assumptions:

         alpha =   0.0500  (two-sided)
            p1 =   0.2000
            p2 =   0.4000
sample size n1 =      155
            n2 =       78
         n2/n1 =     0.50

Estimated power:

         power =   0.8626
```

12. Data collection

Epidemiological measures

In-utero exposures

Maternal prenatal and birth data is available from the larger clinical trial, on gravidity, age, HIV status, use of antimalarial interventions such as bed nets and intermittent preventive therapy (IPTp), socio-economic status, education level, frequency of malarial parasitemia (by RDT, PCR and malaria smears) during pregnancy and placental malaria status at delivery by histopathology.

Infant outcome measures and determinants

Data on infant use of malaria interventions such as bed nets. At scheduled visits, infant use of antimalarial interventions, episodes of illness and overall well-being characterized by serial growth monitoring at under-5 clinic visits will be collected. At unscheduled visits due to illness, the current duration and symptoms of the illness will be recorded and an RDT and haemoglobin measurement shall be done to test for malaria and anaemia as part of point of care.

13. Data management

Managing and preserving data

Data management

Data management support will be done utilizing the available infrastructure and operating procedures under the parent trial, which uses *optical character recognition (OCR)* software for creating and *scanning* (paper-based) data collection forms (*Cardiff TeleForm).* Completed CRFs and relevant source documents will be scanned centrally at the CoM and stored daily (encrypted) on the central server and then batch processed into databases via OCR. CrashPlan-Pro software will ensure automated encrypted back-ups to a second server at the MLW in Blantyre via the local network and off-site in Liverpool via the internet. Databases will be exported to Microsoft Access and SPSS for data manipulation and verification. Data validation and verification will be done to ensure that the data in the database corresponds with the CRFs and source documents. At the end of the study and when the validation

process is concluded the database will be locked. Data analysis will be conducted in SPSS, STATA or SAS.

Archiving

Hard copies of source material (CRF books) will remain on site until completion of field work and stored long term at CoM for a minimum of 5 years. The data will be kept in a secure location and only research staff will have access to the data. Data will also be kept electronically (password protected) at the CoM in compliance with prevailing laws on data storage.

14. Data analysis

Statistical support

Statistical support will be provided by Mr Arthur Kang'ombe and Dr Brian Faragher from LSTM.

Analytical approach

The concept of the analytical approach will be similar among the different types of analyses. Evidence of malaria infection in the pregnant women will be the 'exposure' variable of interest, and neurocognitive (MDAT, A-not-B score, Delay Inhibition, MacArthur Bates) and epidemiological (anaemia, clinical malaria, growth parameter) measures will be the outcome variables.

Bi-variate analysis will be conducted first to explore relationships between exposures and outcomes, followed by multi-variate modelling. Because of the observational nature adjustment of potential confounders will be important.

Frequency and timing of infection (2nd trimester vs 3rd trimester), interventions used (IPTp vs ISTp) will be assessed as exposure variable and/or effect modifiers.

Adjustment for confounding

As an observational study where the 'exposure' variable cannot be allocated at random (e.g. presence of placental infection or malaria during pregnancy), the study is prone to bias. Lower neurocognitive scores or excess malaria in infants (endpoint) born to mothers with

malaria (exposure variable), may simply reflect environmental factors rather than a biological association. The parent trial involves assessment of as many of the potential confounders, including a detailed assessment of the socio-economic parameters, education, net use, other vector control measures such as IRS, various demographic parameters etc.

Bias from the intervention allocation in the parent trial (IPTp vs ISTp) is of less of a concern because the allocation will be random, the endpoints are objective measures (e.g. RDT positivity (with QA by digital photographs) and include preterm birth assessed by ultrasound at enrolment and birth weight. All analysis for the birth cohort study will take treatment allocation during pregnancy into account a priori, either as effect modifier (does the magnitude of association between pre-natal exposure and outcomes of interest depend on the intervention received by the mother during pregnancy?) or as confounder. Clearly if the intervention arm acts as effect modifier, this will have to be taken into account in all further analysis, by stratifying the results by treatment arms.

Planned subgroup analyses

Effect modification will be explored by adding interaction terms in multivariate regression models to explore the effect of season, gravidity, age of child, geographical location (e.g. study site). This will be exploratory in nature, as the study may not have sufficient power for some of these sub-group analyses. All models will include the randomisation groups of the parent either as effect modifier (if there is evidence for effect modification) or as potential confounder.

Statistical methods

The Andersen-Gill extension of Cox regression model for multiple episodes will be used, which is particularly suitable for high incidence diseases such as malaria where the effect of an intervention (ISTp) or other exposure variables (such as placental malaria) on incidence may change during the follow-up time.[33] It is also allows to estimate the direct, indirect and total effects of interventions. The primary analysis will focus on the total effect. All analyses will take the randomization groups into account, either as effect modifier (if there is evidence for effect modification) or as potential confounders.

15. Result presentation and dissemination

The results of the study will be shared early with the NMCR of the MOH and will also be

presented at national and international conferences and submitted for publication in peer-reviewed journals, in accordance with the University Of Malawi College Of Medicine's publication policy.

16. Ethical considerations

Ethical review and research governance arrangements

Ethical clearance will be obtained from Research Ethics Committee of the CoM (CoMReC), University of Toronto, Canada and the University of Liverpool, United Kingdom. Trial authorisation for the parent study has been obtained from the National Health Sciences Research Council of Malawi and the Malawian Pharmacy and Poisons Board. The parent trial has a DSMB. Clinical monitoring of the parent study is conducted by the Research Support Centre of the CoM and all staff are trained in GCP.

Risks and benefits

There are no major risks involved in this study. Participants may feel discomfort at the time when the blood sample is taken. This will be minimized by using experienced nurses and clinicians.

By consenting to take part in this study, children of participants who do not attend scheduled appointment will receive reminders and active follow-up. Participants experiencing illness between visits will be seen and treated free of charge as part of the study; however this is available to all children in Malawi.

Informed consent

Patient information sheets and consent forms in English and Chichewa are attached (Appendix I-VI). The need for all caretakers to fully understand the consent will be made clear to the research team. They have been prepared by a process of translation and back-translation by 2 separate Chichewa-speakers to check for preservation of meaning.

Confidentiality

All counselling will be undertaken confidentially. All hardcopy forms that include patients' information will be carefully stored in a locked cabinet and electronic data will be password protected. Study participant numbers will be assigned sequentially.

Knowledge of mother and infant HIV status

During recruitment of participants, it is possible that some individuals will be HIV positive. The test results will be discussed with the parents or guardians by the research clinicians, who will have completed training in HIV pre- and post-test counselling.

Compliance with guidelines

The study will be conducted in accordance with the 1996 ICH GCP guidelines and the 2000 Declaration of Helsinki, providing each participant with the health care they deserve even if they decide to pull out of the study at any stage and respecting all ethical recommendations.

17. Possible constraints

The study could be limited by a lack of participants after 46 weeks from last study contact. However, based on attrition rates from the parent study, we feel confident that the target numbers will be reached. Retention of participants and compliance to follow-up may additionally pose challenges to the study but use of community contact tracing, as well as reduction in the number and frequency of invasive clinical procedures and visits is anticipated to ameliorate drop outs. In addition, the cordial relationship that the senior Principal Investigator has developed in the area has fostered an amicable working relationship with the community.

18. Management of the Project

Roles investigators and senior support staff

Prof Victor Mwapasa will be the PI on the parent trial and the subsequent birth cohort. He will be supported by Prof Kevin Kain and Prof Feiko ter Kuile from the University of Toronto and Liverpool School of Tropical Medicine respectively as well as Dr Linda Kalilani Phiri. Prof

Kevin Kain will act as chief investigator. Dr Melissa Gladstone and Dr Andrea Conroy will be responsible for the neurocognitive component and Dr Mwayi Madanitsa will be responsible for the onsite clinical and epidemiological coordination of the study. Dr Doreen Ali is based with the National Malaria Control Programme and will be the liaison for the dissemination of the results and possible policy implications at national level. Statistical support will be provided by Dr Brian Faragher and Mr Arthur Kang'ombe at the LSTM.

Legal Sponsorship

The University of Toronto will be the main coordinating institution of this infant project and act as sponsor for the study.

Administration

The College of Medicine will be the main administrator of the grant and responsible for the narrative and financial reporting for study site expenditures

19. Budgetary estimates

**Budget category**	**Total (CA$)**
Personnel	133,250.45
Travel	3,500.00
Direct Supplies and Services	16,249.42
Equipment	23,492.85
Other Research	26,280.00
Sub-grants and Sub-contracts	24,500.00
Total Indirect project costs (CoM Overhead)	22,727.27
**Total Project Costs**	**249,999.99**

20. Justification of the budget

A total budgetary line of CAD 250,000 is required for the conduction of the above study over a period of 18 months.

Personnel

The study will require core funding for 4 research nurses, one study coordinator, 1 data clerk and 1 driver.

Equipment and services

A vehicle (and running costs) is required for the transportation of supplies to the sites as well as site supervision and transporting of participants and staff. A user licence for the Verifier module for Teleform (Optical data capture software) will be required as this will be the rate limiting process in the management of the data. The use of optical data capture to enter data will significantly reduce data entry errors and time thus increasing the accuracy and credibility of the data captured.

Clinical Supplies

Malaria Rapid Diagnostic Tests will be required for testing the infants. Clinical consumables will be required for the provision of safe clinical procedures.

Participant reimbursements

Transport reimbursements will be provided to the participants at both scheduled and unscheduled visits. Community health workers will be used to trace participants who have missed visits. Non-monetary reimbursement (baby soap, laundry soap and nappies) will be required to reimburse the participants for their time.

Office consumables

Office supplies will be required for production of Case Report Forms, study governance documents and document storage as well as financial and technical reports and final study report writing and submission.

21. References

1. Snow RW, Guerra CA, Noor AM, Myint HY, Hay SI, 2005. The global distribution of clinical episodes of Plasmodium falciparum malaria. Nature 434: 214-7.
2. Rogerson SJ, Hviid L, Duffy PE, Leke RF, Taylor DW, 2007. Malaria in pregnancy: pathogenesis and immunity. The Lancet infectious diseases 7: 105-17.
3. Desai M, ter Kuile FO, Nosten F, McGready R, Asamoa K, Brabin B, Newman RD, 2007. Epidemiology and burden of malaria in pregnancy. The Lancet infectious diseases 7: 93-104.
4. Dellicour S, Tatem AJ, Guerra CA, Snow RW, ter Kuile FO, 2010. Quantifying the number of pregnancies at risk of malaria in 2007: a demographic study. PLoS medicine 7: e1000221.
5. Flick K, Scholander C, Chen Q, Fernandez V, Pouvelle B, Gysin J, Wahlgren M, 2001. Role of nonimmune IgG bound to PfEMP1 in placental malaria. Science 293: 2098-100.
6. Brabin BJ, Romagosa C, Abdelgalil S, Menendez C, Verhoeff FH, McGready R, Fletcher KA, Owens S, D'Alessandro U, Nosten F, Fischer PR, Ordi J, 2004. The sick placenta-the role of malaria. Placenta 25: 359-78.
7. Fried M, Nosten F, Brockman A, Brabin BJ, Duffy PE, 1998. Maternal antibodies block malaria. Nature 395: 851-2.
8. Stanton C, Lawn JE, Rahman H, Wilczynska-Ketende K, Hill K, 2006. Stillbirth rates: delivering estimates in 190 countries. Lancet 367: 1487-94.
9. Jaworowski A, Fernandes LA, Yosaatmadja F, Feng G, Mwapasa V, Molyneux ME, Meshnick SR, Lewis J, Rogerson SJ, 2009. Relationship between human immunodeficiency virus type 1 coinfection, anemia, and levels and function of antibodies to variant surface antigens in pregnancy-associated malaria. Clinical and vaccine immunology : CVI 16: 312-9.
10. McDonald CR, Elphinstone RE, Kain KC, 2013. The impact of placental malaria on neurodevelopment of exposed infants: a role for the complement system? Trends Parasitol 29: 213-9.
11. Conroy AL, McDonald CR, Silver KL, Liles WC, Kain KC, 2011. Complement activation: a critical mediator of adverse fetal outcomes in placental malaria? Trends Parasitol 27: 294-9.
12. Hviid L, 2009. Unraveling the impact of malaria exposure before birth. PLoS medicine 6: e1000117.
13. Rogerson SJ, 2010. Malaria in pregnancy and the newborn. Advances in experimental medicine and biology 659: 139-52.
14. Hartman TK, Rogerson SJ, Fischer PR, 2010. The impact of maternal malaria on newborns. Annals of tropical paediatrics 30: 271-82.
15. ter Kuile FO, van Eijk AM, Filler SJ, 2007. Effect of sulfadoxine-pyrimethamine resistance on the efficacy of intermittent preventive therapy for malaria control during pregnancy: a systematic review. JAMA 297: 2603-16.
16. Feng G, Simpson JA, Chaluluka E, Molyneux ME, Rogerson SJ, 2010. Decreasing burden of malaria in pregnancy in Malawian women and its relationship to use of intermittent preventive therapy or bed nets. PLoS One 5: e12012.
17. Harrington WE, Mutabingwa TK, Muehlenbachs A, Sorensen B, Bolla MC, Fried M, Duffy PE, 2009. Competitive facilitation of drug-resistant Plasmodium falciparum malaria parasites in pregnant women who receive preventive treatment. Proc Natl Acad Sci U S A 106: 9027-32.
18. Tagbor H, Bruce J, Agbo M, Greenwood B, Chandramohan D, 2010. Intermittent screening and treatment versus intermittent preventive treatment of malaria in pregnancy: a randomised controlled non-inferiority trial. PloS one 5: e14425.
19. Smith LA, Jones C, Adjei RO, Antwi GD, Afrah NA, Greenwood B, Chandramohan D, Tagbor H, Webster J, 2010. Intermittent screening and treatment versus intermittent preventive treatment of malaria in pregnancy: user acceptability. Malaria journal 9: 18.

20. Le Hesran JY, Cot M, Personne P, Fievet N, Dubois B, Beyeme M, Boudin C, Deloron P, 1997. Maternal placental infection with Plasmodium falciparum and malaria morbidity during the first 2 years of life. American journal of epidemiology 146: 826-31.
21. Cot M, Le Hesran JY, Staalsoe T, Fievet N, Hviid L, Deloron P, 2003. Maternally transmitted antibodies to pregnancy-associated variant antigens on the surface of erythrocytes infected with Plasmodium falciparum: relation to child susceptibility to malaria. American journal of epidemiology 157: 203-9.
22. Mutabingwa TK, Bolla MC, Li JL, Domingo GJ, Li X, Fried M, Duffy PE, 2005. Maternal malaria and gravidity interact to modify infant susceptibility to malaria. PLoS medicine 2: e407.
23. Kalanda BF, Verhoeff FH, Chimsuku L, Harper G, Brabin BJ, 2006. Adverse birth outcomes in a malarious area. Epidemiology and infection 134: 659-66.
24. Watson-Jones D, Weiss HA, Changalucha JM, Todd J, Gumodoka B, Bulmer J, Balira R, Ross D, Mugeye K, Hayes R, Mabey D, 2007. Adverse birth outcomes in United Republic of Tanzania--impact and prevention of maternal risk factors. Bulletin of the World Health Organization 85: 9-18.
25. Verhoeff FH, Le Cessie S, Kalanda BF, Kazembe PN, Broadhead RL, Brabin BJ, 2004. Post-neonatal infant mortality in Malawi: the importance of maternal health. Annals of tropical paediatrics 24: 161-9.
26. Malhotra I, Dent A, Mungai P, Wamachi A, Ouma JH, Narum DL, Muchiri E, Tisch DJ, King CL, 2009. Can prenatal malaria exposure produce an immune tolerant phenotype? A prospective birth cohort study in Kenya. PLoS medicine 6: e1000116.
27. Schwarz NG, Adegnika AA, Breitling LP, Gabor J, Agnandji ST, Newman RD, Lell B, Issifou S, Yazdanbakhsh M, Luty AJ, Kremsner PG, Grobusch MP, 2008. Placental malaria increases malaria risk in the first 30 months of life. Clinical infectious diseases : an official publication of the Infectious Diseases Society of America 47: 1017-25.
28. Adegnika AA, Kohler C, Agnandji ST, Chai SK, Labuda L, Breitling LP, Schonkeren D, Weerdenburg E, Issifou S, Luty AJ, Kremsner PG, Yazdanbakhsh M, 2008. Pregnancy-associated malaria affects toll-like receptor ligand-induced cytokine responses in cord blood. The Journal of infectious diseases 198: 928-36.
29. Bonner PC, Zhou Z, Mirel LB, Ayisi JG, Shi YP, van Eijk AM, Otieno JA, Nahlen BL, Steketee RW, Udhayakumar V, 2005. Placental malaria diminishes development of antibody responses to Plasmodium falciparum epitopes in infants residing in an area of western Kenya where P. falciparum is endemic. Clinical and diagnostic laboratory immunology 12: 375-9.
30. Gladstone M, Lancaster GA, Umar E, Nyirenda M, Kayira E, van den Broek NR, Smyth RL, 2010. The Malawi Developmental Assessment Tool (MDAT): the creation, validation, and reliability of a tool to assess child development in rural African settings. PLoS Med 7: e1000273.
31. Caldwell BM, Bradley RH, 1979. Home Observation for Measurement of the Environment (HOME). Little Rock: University of Arkansas.
32. Fenson L, Pethick S, Renda C, Cox J, Dale P, Reznick JS, 2000. Short-form versions of the MacArthur Communicative Development Inventories. Applied Psycholinguistics 21: 95-116.
33. Cheung YB, Xu Y, Tan SH, Cutts F, Milligan P, 2010. Estimation of intervention effects using first or multiple episodes in clinical trials: The Andersen-Gill model re-examined. Statistics in medicine 29: 328-36.
34. Holding PA, Snow RW: Impact of Plasmodium falciparum malaria on performance and learning: review of the evidence. Am J Trop Med Hyg 2001, 64:68-75
35. Fernando et al: The 'hidden' burden of malaria: cognitive impairment following infection. Malaria Journal 2010 9:366

36. Fink et al.: Association between early childhood exposure to malaria and children's pre-school development: evidence from the Zambia early childhood development project. Malaria Journal 2013 12:12

37. Mwaniki MK, Atieno M, Lawn JE, Newton C: Long-term neurodevelopmental outcomes afterintrauterine and neonatal insults: a systematic review. Lancet 2012; 379:445-52

22. APPENDIX I (English Information Sheet)

What is the purpose of the study?

Babies who are born to mothers who have had malaria during their pregnancy may have reduced intelligence than babies who are born to mothers who did not have malaria during pregnancy. We would like to find out why and how this happens. In order to achieve this we would like to compare the development of intelligence of babies born to women how live in areas which have high malaria infections.

Why has your child been chosen?

Your child has been chosen to participate in this study because he/she has been born to a mother who participated in a study of pregnant women who live in areas with a lot of malaria.

Is your child eligible to participate in the study?

Your child can participate in this study if he/she:

a. Is not participating in any other study
b. Is otherwise healthy

Your child cannot participate in this study if your child:

a. Is unwell
b. Will not be available for follow up for a year within the study catchment area

Does your child have to take part in the study?

No, the study is voluntary, and your child does not have to participate. If you change your mind later, you can withdraw your child from the study without giving any reason.

What is expected if you agree to have your child participate in the study?

We will test the child's intelligence at 12, 18 and 24 months of age. We will also test your child for malaria and treat them if they are found with malaria.

Will there be any risks involved in the study?

There are no special risks apart from taking blood to test for malaria when your child is sick. The needle may cause little pain and occasionally mild bruising.

Will there be any benefits involved in the study?

If your child if found to have malaria parasites in his blood, we will provide him with anti-malarial drugs. The study will not interrupt with your child's under-five health care at any time.

Can I withdraw my child from the study at any time?

Yes, you can withdraw your child from the study at any stage and you do not need to give a reason.

Who is organising the study?

The study is being organised by Dr Mwayiwawo Madanitsa of the College of Medicine, and has been approved by the College of Medicine Research Ethics Committee. If you have any questions concerning the study, you may contact Dr Mwayiwawo Madanitsa on 0111619209 or the College of Medicine Research Ethics Committee secretariat on 01 877 245.

Will the information obtained in the study be confidential?

Yes, all information will be kept confidential. No one can see the information or results except the staff running the study. The information will be kept safely for 5 years, and then it will be destroyed. The overall results will be published so they can be used to improve our understanding of the effect of malaria in pregnancy on the development of a child's intelligence and what can be done to protect the development of the intelligence of children born and living in areas with a lot of malaria.

23. APPENDIX II (English Consent Form)

Participant consent form

Name...

Address...
....

1 Have you read or listened to the participant information sheet? Yes / No

2 Have you had the opportunity to ask questions? Yes / No

3 Have your questions been answered, and do you feel that you have had enough information about this study? Yes / No

4 Do you understand that your child is free to withdraw from the study at any time without giving a reason and without affecting your future care at this hospital? Yes / No

If you have answered 'yes' to these questions, please sign the form, or place a thumbprint below, which means that you agree to allow your child to enter the study.

I voluntarily agree/ disagree for my baby to participate in this research.

Signature... Date …………………………..

Staff obtaining consent (name in capitals)…………………………………………….

Signature .... Date …………………………

Impartial witness (where needed)

Signature .... Date …………………………

24. APPENDIX III (Chichewa Information Sheet)

Kodi cholinga cha Kafuku-fukuyu ndi chiyani?

Ana omwe amabadwa kwa azimayi amene anadwala malungo panthawi yomwe alindimimba amakhoza kukhala ndinzelu zochepa poyelekeza ndi ana amene amabadwa kwa amai amene sanadwale malungo panthawi yomwe amayembekezela. Ife tikufuna kuti tifufuze chomwe chimachititsa zimenezi komanso zimachitika bwanji. Kuti ife tikwanilitse kafukufuku ameneyu pakufunika kuti tifananitse ndikusiyanitsa m'mene nzelu za ana obadwa mumadela amene malungo ndiochuluka, ama kula ndi kukhwima.

Ndichifukwa chiyani mwana wanu wasankhidwa?

Mwana wanu wasankhidwa kutengapo gawo pa kafukufuku ameneyu chifukwa choti iyeyu anabadwa kwamayi amene anatenganawo mbali mukafuku-fuku wa amayi oyembekezela mudela limene lilindi malungo ochuluka.

Kodi mwana wanu ndiwoyenera kutenga mbali?

Mwana wanu ndiwoyenera kutenga mbali ngati:

a. Asakutengapo mbali mukafukufuku wina
b. Ali wathanzi

Mwana wanu sangathe kutenga mbali mu kafuku-fukuyu ngati:

a. Ali wodwala
b. Sakhala mudela yozungulira mzinda umene kafuku-fukuyu ukuchitikila kwachaka chimodzi.

Kodi mwana wanga akuwumilizidwa kutenga mbali mu kafuku-fukuyu?

Ayi, kafuku-fukuyu munthu asamuumilize mwana wanu kutenga mbali koma atenge mbali ngati inuyo monga kholo mwafuna ndipo mutasintha maganizo angathe kusiya ngakhale osapereka chifukwa.

Kodi nditabvomera kuti mwana wanga atenge mbali mu kafuku-fukuyu kudzachitika zotani?

Tidzayeza kukula ndi kukhwima kwa nzelu za mwana wanu akakhala ndichaka chimodzi, chaka chimodzi nditheka komanso akakwanitsa zaka ziwili. Tidzamuyezaso mwanuyo malungo ngati agapezeke kuti sakupeza bwino muthupi.

Kodi pali zowopsya zanji mu kafuku-fuku ameneyu?

Palibe choopsya chirichonse kupatula kutenga magazi panthawi imen mwana wanu angadwale, kuti ayezedwe malungo . Singano nthawi zina imawawa kapenanso kubweretsa kachilonda kakang'ono.

Kodi pali phindu lirilonse mu kafuku-fuku ameneyu?

Inde phindu liripo chifukwa choti ngati mwana wanu adzapezeka kuti ali ndi t malungo, ife tidzamupatsa mankhwala a malungo. Kafukufukuwu sazasokoneza ndondomeko yakhusikelo yamwana wanu.

Kodi mwana wanga angathe kutuluka mu kafuku-fukuyu nthawi iliyonse?

Inde, mwana wanu akhonza kutuluka mukafukufukuyi nthawi ina ili yonse ngakhale osapereka chifukwa china chilichonse.

Kodi yemwe akukonza kafuku-fukuyu ndi ndani?

Kafuku-fukuyu wakonzedwa ndi Dr Mwayiwawo Madanitsa aku College of Medicine.Owona za kafuku-fuku a kusukulu yaukachenjede ya madokotala (COMREC) apereka chilolezo kuti kafuku-fukuyu achitidwe. Ngati muli ndi funso lina lililonse lokhudzana ndi kafukufukuyi mikhoza kulankhula ndi Dr Mwayiwawo Madanitsa pa nambala yatelefoni iyi 0111619209 kapena ndi aCOMREC pa nambala yatelefoni iyi:01 877 245.

Kodi zomwe adzapeze mu kafuku-fukuyu zidzakhala za chinsinsi?

Inde zonse zidzasungidwa mwa chinsinsi. Palibe yemwe adzaone zolembedwa kapena zotsatira kupatula eni ake omwe adzidzagwira ntchitoyi. Zonse zidzasungidwa mwa chinsinsi kwa zaka zisanu kenako zidzaonongedwa. Zotsatira zidzalembedwa kuti zithandize kuunikila m'mene mwana wosabadwa amakhudzidwa mukukula ndi kukhwima kwanzelu zake, mai akadwala malungo ali oyembekezela komaso kuunikila njila zimene

zingathadize kuteteza kukula ndi kukhwima kwa nzelu za ana obadwa ndi kukhala mumadela amene alindi malungo ochuluka.

25. APPENDIX IV (Chichewa Consent Form)

Fomu yovomeleza mwana wanu kutenganawo mbali mu kafuku-fuku ameneyu.

Dzina...

Khela...

....

1 Mwawelenga kapena kumvetsera fomu yolongosola kufuku-fuku? Inde / Ayi

2 Mwakhala ndimpata wofunsa mafunso ? Inde / Ayi

3 Kodi mafunso anu ayankhidwa ndipo mwvetsetsa zolinga

ndizochitika zakafuku-fuku ? Inde / Ayi

4 Mwamvetsetsa kuti muliomasukha kutulutsa mwana wanu mukafuku-fukuyi nthawi ina ili yonse ngakhale osapereka chifukwa chilichonse? Inde / Ayi

Ngati mwayankha 'Inde' kumafunso onsewa, chonde sayinani fomu iyi kapena dhindhani chalachachikulu potsimikiza kuti mwavetsetsa zakafuku-fukuyi ndi kuvomela kuti mwana wanu antengenawo mbali mukafuku-fukuyi.

Ine, mosaumilizidwa, ndikuvomeleza / sindikuvomeleza kuti mwana wanga atengenawo mbali mukafukufukuwu.

Sayini... Tsiku …………………………..

Wakafuku-fuku wotenga chivomelo (dzina mumalemba akulu)…………………………………..

Sayini .... Tsiku …………………………

Mboni osatengera mbali (ngati kholo samatha kuwelenga)

Sayini .... Tsiku …………………………
