# Supplementary material for: Neurocognitive outcomes in Malawian children exposed to malaria during pregnancy: An observational birth cohort study
Source: PLoS Med. 2021 Sep 28;18(9):e1003701. doi: 10.1371/journal.pmed.1003701 (PMC8478258; doi:10.1371/journal.pmed.1003701)
Supplement: S1 Table — (DOCX) [file pmed.1003701.s004.docx]

| **Supplementary Table 1. Raw neurocognitive scores at 18 and 24 months by maternal malaria status during pregnancy** | | | | | | | | | |
| --- | --- | --- | --- | --- | --- | --- | --- | --- | --- |
|  | | | | | | | | | |
|  | **Malaria Exposure^b^** | **18 months** | | | | **24 months** | | | |
| **Outcome^a^** |  | **n/N (%)^c^** | **Malaria**  **negative** | **Malaria**  **positive** | **p-value^d^** | **n/N (%)^c^** | **Malaria**  **negative** | **Malaria**  **positive** | **p-value^d^** |
| A-not-B^e^ | Antenatal | 201/357 (56.3) | 80 [60, 100] | 80 [60, 100] | 0.487 | 183/336 (54.5) | 100 [80, 100] | 100 [80, 100] | 0.896 |
|  | *14–23* | 123/293 (42.0) | 80 [60, 100] | 80 [60, 100] | 0.438 | 109/273 (39.9) | 100 [80, 100] | 100 [80, 100] | 0.989 |
|  | *>23–28* | 60/325 (18.5) | 80 [60, 100] | 80 [57, 100] | 0.792 | 57/308 (18.5) | 100 [80, 100] | 100 [80, 100] | 0.226 |
|  | *>28–33* | 62/348 (17.8) | 80 [60, 100] | 90 [60, 100] | 0.436 | 65/329 (19.8) | 100 [80, 100] | 100 [80, 100] | 0.567 |
|  | *> 33-37* | 50/293 (17.1) | 80 [60, 100] | 80 [58, 100] | 0.586 | 53/274 (19.3) | 100 [80, 100] | 100 [80, 100] | 0.660 |
|  | Placental | 96/319 (30.1) | 80 [60, 100] | 80 [57, 100] | 0.965 | 87/305 (28.5) | 100 [80, 100] | 100 [80, 100] | 0.925 |
|  |  |  |  |  |  |  |  |  |  |
| Delayed Inhibition  (Sweet)^f^ | Antenatal | 196/357 (54.9) | 111/161 (68.6) | 138/196 (70.4) | 0.765 | 177/328 (54.0) | 62/151 (41.1) | 82/177 (46.3) | 0.341 |
|  | *14–23* | 119/291 (40.9) | 120/172 (69.8) | 82/119 (68.9) | 0.876 | 103/264 (39.0) | 69/161 (42.9) | 47/103 (45.6) | 0.656 |
|  | *>23–28* | 60/325 (18.5) | 185/265 (69.8) | 43/60 (71.7) | 0.772 | 58/301 (19.3) | 103/243 (42.4) | 30/58 (51.7) | 0.176 |
|  | *>28–33* | 65/349 (18.6) | 196/284 (69.0) | 46/65 (70.8) | 0.778 | 66/321 (20.6) | 106/255 (41.6) | 34/66 (51.5) | 0.127 |
|  | *> 33-37* | 52/292 (17.8) | 167/240 (70.0) | 34/52 (65.4) | 0.570 | 53/269 (19.7) | 88/216 (40.7) | 25/53 (47.2) | 0.380 |
|  | Placental | 95/319 (29.8) | 158/224 (70.5) | 66/95 (69.5) | 0.851 | 82/300 (27.3) | 88/218 (40.4) | 41/82 (50.0) | 0.120 |
|  |  |  |  |  |  |  |  |  |  |
| Delayed Inhibition (Biscuit)^f^ | Antenatal | 196/357 (54.9) | 94/161 (58.4) | 119/196 (60.7) | 0.656 | 177/328 (54.0) | 45/151 (29.8) | 46/177 (26.0) | 0.442 |
|  | *14–23* | 119/291 (40.9) | 103/172 (59.9) | 73/119 (61.3) | 0.801 | 103/264 (39.0) | 41/161 (25.5) | 30/103 (29.1) | 0.511 |
|  | *>23–28* | 60/325 (18.5) | 157/265 (59.2) | 38/60 (63.3) | 0.547 | 58/301 (19.3) | 68/243 (28.0) | 12/58 (20.7) | 0.275 |
|  | *>28–33* | 65/349 (18.6) | 171/284 (60.2) | 36/65 (55.4) | 0.491 | 66/321 (20.6) | 72/255 (28.2) | 17/66 (25.8) | 0.692 |
|  | *> 33-37* | 52/292 (17.8) | 144/140 (60.0) | 29/52 (55.8) | 0.586 | 53/269 (19.7) | 62/216 (28.7) | 14/53 (26.4) | 0.743 |
|  | Placental | 95/319 (29.8) | 133/224 (59.4) | 62/95 (65.3) | 0.310 | 82/300 (27.3) | 58/218 (26.7) | 25/82 (30.5) | 0.498 |
| ^a^ Age of A-not-B and Delayed Inhibition first assessment was 18 months. ^b^Malaria exposures defined as antenatal malaria (peripheral PCR-confirmed malaria at any point during pregnancy) or placental malaria (positive placental histology or placental PCR at delivery). The former is stratified by gestational age (*weeks*) at the time of PCR-confirmed infection.  ^c^n/N(%): malaria positive women as a percent of total women (N) with existing data for both the respective neurocognitive score and malaria variable. ^d^p-values represent unadjusted weighted quasibinomial regression for A-not-B (weighted for the number of completed trials), and unadjusted log-binomial regression for Delayed Inhibition, before correction for multiple comparisons. No p-values were significant after Holm-Bonferonni adjustment for multiple comparisons across age at neurocognitive assessment and malaria exposure variables (n=12). ^e^Scores are presented as the proportion of completed trials that were correct (expressed as median [IQR]). Only n=257 and n=264 children completed all 10 trials at 18 and 24 months of age, respectively.  ^f^Scores are presented as the number of incomplete delays in the respective exposure group (expressed as n/N exposure group [%]). | | | | | | | | | |
