# Supplementary material for: Neurocognitive outcomes in Malawian children exposed to malaria during pregnancy: An observational birth cohort study
Source: PLoS Med. 2021 Sep 28;18(9):e1003701. doi: 10.1371/journal.pmed.1003701 (PMC8478258; doi:10.1371/journal.pmed.1003701)
Supplement: S2 Table — (DOCX) [file pmed.1003701.s005.docx]

| **Supplementary Table 2. Detailed breakdown of malaria infections in maternal cohort** | |
| --- | --- |
| Characteristics | n (%)^a^ |
| **Antenatal Malaria^b^** |  |
| *n* positive PCR tests: |  |
| 0 | 179 (42.7) |
| 1 | 118 (28.2) |
| 2-3 | 96 (22.9) |
| 4-6 | 26 (6.2) |
| *n* positive PCR by gestational age: |  |
| 14–23 weeks | 147 (42.5) |
| > 23–28 weeks | 75 (19.6) |
| > 28–33 weeks | 79 (19.4) |
| > 33–37 weeks | 62 (17.9) |
| Delivery | 58 (15.1) |
| **Placental Malaria** |  |
| Placental Histology | 79 (21.1) |
| Past (% of positive) | 43 (54.4) |
| Active (% of positive) | 36 (45.6) |
| Chronic (% Active) | 31 (86.1) |
| Acute (% Active) | 5 (13.9) |
| Placental PCR | 58 (16.1) |
| Antenatal and Placental Malaria | 91 (21.6) |
| ^a^n (%) of women with existing data for that variable. Proportion of missing data presented in S5 Table. ^b^Peripheral PCR-positive malaria across pregnancy, including enrolment and delivery. | |
