# Supplementary material for: Neurocognitive outcomes in Malawian children exposed to malaria during pregnancy: An observational birth cohort study
Source: PLoS Med. 2021 Sep 28;18(9):e1003701. doi: 10.1371/journal.pmed.1003701 (PMC8478258; doi:10.1371/journal.pmed.1003701)
Supplement: S3 Table — (DOCX) [file pmed.1003701.s006.docx]

| **Supplementary Table 3. MDAT and MCAB-CDI at 12, 18, and 24 months by cord-blood PCR malaria status** | | | | | | | | | |
| --- | --- | --- | --- | --- | --- | --- | --- | --- | --- |
|  | **12 months** | | | **18 months** | | | **24 months** | | |
|  | **CB negative** | **CB positive** | **p-value^a^** | **CB negative** | **CB positive** | **p-value^a^** | **CB negative** | **CB positive** | **p-value^a^** |
| **CB PCR malaria, n (%)^b^** | 109 (90.1) | 12 (9.9) |  | 281 (90.1) | 31 (9.9) |  | 266 (90.8) | 27 (9.2) |  |
| **Primary Outcome (Total Score)^c^** | | | | | | | | | |
| MCAB-CDI^d^ | **---** | **---** | **---** | 35.1 (23.7) | 34.4 (22.9) | 0.875 | 75.2 (22.5) | 78.8 (17.8) | 0.405 |
| MDAT Total | 58.9 (5.0) | 58.5 (4.9) | 0.792 | 70.9 (5.1) | 72.7 (5.8) | 0.073 | 89.5 (6.9) | 89.1 (6.6) | 0.770 |
| **Secondary Outcome (Subdomain Score)^c^** | | | | | | | | | |
| MDAT Gross Motor | 16.1 (1.6) | 16.6 (2.1) | 0.283 | 20.2 (1.5) | 20.4 (1.9) | 0.715 | 21.5 (1.4) | 21.1 (1.3) | 0.170 |
| MDAT Fine Motor | 16.4 (1.7) | 16.8 (1.1) | 0.442 | 19.2 (1.4) | 19.8 (1.4) | 0.040 | 23.4 (1.9) | 23.2 (2.1) | 0.515 |
| MDAT Language | 10.5 (2.3) | 9.7 (1.6) | 0.218 | 12.7 (1.9) | 13.5 (2.2) | 0.037 | 18.8 (3.1) | 18.7 (3.1) | 0.972 |
| MDAT Social | 15.9 (1.8) | 15.4 (1.8) | 0.413 | 18.7 (2.5) | 19.1 (2.6) | 0.508 | 25.9 (2.8) | 26.1 (2.6) | 0.658 |
| ^a^p-value represents unadjusted ordinary least squares regression. No p-values are significant after Holm-Bonferroni adjustment for multiple comparisons across age at assessment (n=3 for MDAT; n=2 for MCAB-CDI).  ^b^n(%) of women with cord blood data whose children also have an MDAT score. n(%) of women whose children also have an MCAB-CDI score at 18 months = 30 (10.6) and at 24 months = 30 (9.7). ^c^Scores represented as mean (standard deviation). ^d^Age of MCAB-CDI first assessment was 18 months. Abbreviations: Cord blood (CB), Malawi Development Assessment Tool (MDAT), McArthur Bates Communication Development Inventory (MCAB-CDI). | | | | | | | | | |
