# Supplementary material for: Neurocognitive outcomes in Malawian children exposed to malaria during pregnancy: An observational birth cohort study
Source: PLoS Med. 2021 Sep 28;18(9):e1003701. doi: 10.1371/journal.pmed.1003701 (PMC8478258; doi:10.1371/journal.pmed.1003701)
Supplement: S4 Table — (DOCX) [file pmed.1003701.s007.docx]

| **Supplementary Table 4. Descriptive characteristics of PAMaNeD study population by parent trial treatment arm** | | | | | | | |
| --- | --- | --- | --- | --- | --- | --- | --- |
|  | | | | | **IPTp-SP** | **ISTp-DP** |  |
| n | | | | | 218 | 203 |  |
| **Baseline Characteristics** | | | | | **n (%)^a^ or median [IQR]** | | **p-value^b^** |
| Maternal age (years) | | | | | 21 [19, 25] | 21 [19, 26] | 0.555 |
| Gestational age at enrolment (weeks) | | | |  | 19.9 [17.9, 22.1] | 19.6 [17.8, 22.0] | 0.380 |
| Socioeconomic status (tertile) | | | | 1 | 61 (28.1) | 64 (31.7) | 0.563 |
|  | | | | 2 | 80 (36.9) | 65 (32.2) |  |
|  | | | | 3 | 76 (35.0) | 73 (36.1) |  |
| Hemogloblin at enrolment (g/dL) | | | | | 11.0 [9.9, 12.0] | 10.9 [10.0, 12.0] | 0.473 |
| Primigravidity | | | | | 73 (33.5) | 71 (35.0) | 0.827 |
| Maternal education status (tertile) | | | 1 | | 67 (30.9) | 58 (28.7) | 0.497 |
|  | | | 2 | | 113 (52.1) | 116 (57.4) |  |
|  | | | 3 | | 37 (17.1) | 28 (13.9) |  |
| Family care indicators | | | 12 Months | | 6 [5, 6] | 5 [5, 6] | 0.983 |
|  | | | 18 Months | | 6 [5, 7] | 6 [5, 7] | 0.698 |
|  | | | 24 Months | | 7 [6, 8] | 7 [6, 8] | 0.482 |
| **Perinatal Characteristics** | | | | | | | |
| Gestational age at delivery (weeks) | | | | | 38.7 [37.6, 40.0] | 38.6 [37.4, 39.6] | 0.116 |
| Birth weight (kg) | | | | | 3.0 [2.7, 3.2] | 3.0 [2.7, 3.2] | 0.532 |
| Sex | | Male | | | 115 (53.0) | 96 (47.3) | 0.307 |
|  | | Female | | | 103 (47.2) | 107 (52.7) |  |
| Low birth weight (< 2.5kg) | | | | | 14 (6.5) | 14 (7.2) | 0.953 |
| Preterm Birth (< 37 weeks gestation) | | | | | 32 (14.7) | 39 (19.2) | 0.267 |
| Small-for-gestational age | | | | | 16 (7.4) | 17 (8.7) | 0.781 |
| **Maternal Malaria Status** | | | | | | | |
| Antenatal malaria positive | | | | | 114 (52.5) | 126 (62.4) | 0.053 |
| Placental malaria positive | | | | | 53 (24.3) | 59 (29.1) | 0.272 |
| **Child Malaria Infections^c^** | No Infections | | | | 96 (44.4) | 79 (39.5) | 0.593 |
| One Infection | | | | | 56 (25.9) | 57 (28.5) |  |
| > One Infection | | | | | 64 (29.6) | 64 (32.0) |  |
| **Neurocognitive Outcomes** | | | | | **N (obs)**^d^ | **Estimate (95% CI)** | **p-value^e^** |
| MCAB-CDI | | | | | 407 (695) | 2.22 (-1.84, 6.28) | 0.283 |
| MDAT | | | | | 415 (840) | -0.06 (-0.97, 0.84) | 0.888 |
| ^a^ n(%) expressed as the percentage of women with existing data for the respective variable. ^b^p-value of Chi-square, or Wilcoxon rank-sum test. ^c^Number of malaria infections reported in child's health passport up to 24 months. ^d^Number of subjects (number of observations) included in the mixed models. ^e^p-value of linear mixed-effects likelihood ratio test; reference level of treatment group is IPTp. Abbreviations: Intermittent Screening and Treatment in Pregnancy with Dihydroartemisinin-Piperaquine (IPTp-DP), Intermittent Preventative Treatment in Pregnancy with Sulfadoxine-Pyrimethamine (ISTp-SP), Interquartile Range [IQR], McArthur Bates Communication Development Inventory (MCAB-CDI), Malawi Development Assessment Tool (MDAT), The Effect of Pregnancy Associated Malaria on Early Childhood Neurocognitive Development: an Observational Birth Cohort Study (PAMaNeD). | | | | | | | |
