# Supplementary material for: Neurocognitive outcomes in Malawian children exposed to malaria during pregnancy: An observational birth cohort study
Source: PLoS Med. 2021 Sep 28;18(9):e1003701. doi: 10.1371/journal.pmed.1003701 (PMC8478258; doi:10.1371/journal.pmed.1003701)
Supplement: S5 Table — (DOCX) [file pmed.1003701.s008.docx]

| **Supplementary Table 5. Summary of missing and available data** | |
| --- | --- |
| **Variable** | **n (%) missing for cohort** |
| **Malaria Exposure Variables** |  |
| Antenatal malaria | 2 (0.5) |
| Placental malaria | 43 (10.2) |
| Cord blood malaria | 65 (15.4) |
| Child malaria infection | 5 (1.2) |
| **Covariates** |  |
| Maternal age | 0 (0) |
| Socioeconomic status | 2 (0.5) |
| Treatment group | 0 (0) |
| Birth weight | 12 (2.9) |
| Gestational age at delivery | 0 (0) |
| Sex | 0 (0) |
| Family care indicators (FCI)^a^ |  |
| 12 Months | 2 (1.4) |
| 18 Months | 20 (5.0) |
| 24 Months | 19 (5.1) |
| Age^a^ |  |
| 12 Months | 7 (5.0) |
| 18 Months | 0 (0) |
| 24 Months | 1 (0.3) |
| MUAC^a,b^ |  |
| 12 Months | 20 (14.4) |
| 18 Months | 84 (22.0) |
| 24 Months | 82 (22.1) |
| **Neurocognitive Scores** | **n (%) available for cohort** |
| MDAT |  |
| 12 Months | 139 (33.0) |
| 18 Months | 369 (87.6) |
| 24 Months | 339 (80.5) |
| MCAB |  |
| 18 Months | 333 (79.1) |
| 24 Months | 363 (86.2) |
| ^a^FCI, age, and MUAC are presented as n (%) of children that were missing data but had available neurocognitive scores at that time point. ^b^MUAC was not included as a covariate in multivariate models due to the large amount of missing data. | |
