# Supplementary material for: Neurocognitive outcomes in Malawian children exposed to malaria during pregnancy: An observational birth cohort study
Source: PLoS Med. 2021 Sep 28;18(9):e1003701. doi: 10.1371/journal.pmed.1003701 (PMC8478258; doi:10.1371/journal.pmed.1003701)
Supplement: S6 Table — (DOCX) [file pmed.1003701.s009.docx]

| **Supplementary Table 6. Raw MCAB-CDI and MDAT scores (mean, SD) at 12, 18, and 24 months by maternal malaria status during pregnancy** | | | | | | | | | | | | | |
| --- | --- | --- | --- | --- | --- | --- | --- | --- | --- | --- | --- | --- | --- |
|  | **Malaria Exposure^a^** | **12 months** | | | | **18 months** | | | | **24 months** | | | |
| **Outcome** |  | **n/N (%)^b^** | **Malaria**  **negative** | **Malaria positive** | **p-value^c^** | **n/N (%)^b^** | **Malaria**  **negative** | **Malaria**  **positive** | **p-value^c^** | **n/N (%)^b^** | **Malaria**  **negative** | **Malaria**  **positive** | **p-value^c^** |
| **Total Scores^d^** | | | | | | | | | | | | | |
| MCAB-CDI^e^ | Antenatal | .. | .. | .. | .. | 185/332 (55.7) | 36.4 (24.5) | 33.9 (22.7) | 0.349 | 196/362 (54.1) | 75.7 (21.7) | 75.6 (22.3) | 0.950 |
|  | *14–23* | .. | .. | .. | .. | 115/279 (41.2) | 34.3 (24.1) | 34.7 (23.4) | 0.878 | 118/294 (40.1) | 75.6 (22.0) | 75.4 (22.6) | 0.933 |
|  | *>23–28* | .. | .. | .. | .. | 57/303 (18.8) | 35.4 (23.2) | 34.3 (22.6) | 0.759 | 62/331 (18.7) | 76.2 (21.2) | 75.1 (24.7) | 0.707 |
|  | *>28–33* | .. | .. | .. | .. | 63/323 (19.5) | 35.3 (24.0) | 33.0 (20.9) | 0.484 | 66/352 (18.8) | 76.1 (21.6) | 74.4 (24.1) | 0.565 |
|  | *> 33-37* | .. | .. | .. | .. | 50/278 (18.0) | 37.3 (23.5) | 26.9 (20.2) | 0.004* | 56/296 (18.9) | 78.3 (19.9) | 71.8 (22.8) | 0.033 |
|  | Placental | .. | .. | .. | .. | 88/299 (29.4) | 36.0 (24.3) | 31.8 (21.1) | 0.152 | 94/327 (28.7) | 76.4 (21.1) | 72.3 (24.2) | 0.133 |
|  |  |  |  |  |  |  |  |  |  |  |  |  |  |
| MDAT Total Score | Antenatal | 83/138 (60.1) | 58.8 (4.0) | 58.7 (5.6) | 0.858 | 205/367 (55.9) | 70.7 (5.0) | 71.2 (5.4) | 0.346 | 189/338 (55.9) | 89.5 (7.0) | 89.6 (6.6) | 0.877 |
|  | *14–23* | 57/124 (46.0) | 58.9 (4.2) | 58.3 (6.0) | 0.563 | 127/303 (41.9) | 70.7 (4.8) | 71.4 (5.7) | 0.232 | 112/274 (40.9) | 89.8 (6.6) | 89.8 (6.9) | 0.997 |
|  | *>23–28* | 26/124 (21.0) | 58.4 (4.6) | 59.6 (4.9) | 0.242 | 62/331 (18.7) | 70.9 (5.0) | 71.8 (6.1) | 0.194 | 59/310 (19.0) | 89.6 (6.6) | 89.3 (7.8) | 0.765 |
|  | *>28–33* | 25/137 (18.2) | 58.7 (5.0) | 58.5 (5.0) | 0.842 | 67/358 (18.7) | 70.8 (5.2) | 71.5 (5.4) | 0.344 | 65/331 (19.6) | 89.3 (6.7) | 90.7 (7.1) | 0.129 |
|  | *> 33-37* | 17/117 (14.5) | 58.8 (4.6) | 58.5 (7.3) | 0.798 | 56/305 (18.4) | 71.2 (4.9) | 70.4 (5.9) | 0.290 | 53/276 (19.2) | 90.2 (6.4) | 88.8 (6.2) | 0.175 |
|  | Placental | 34/128 (26.6) | 59.1 (5.5) | 57.3 (3.7) | 0.077 | 98/330 (29.7) | 70.9 (4.9) | 71.4 (5.8) | 0.454 | 91/307 (29.6) | 89.9 (6.7) | 88.9 (7.2) | 0.240 |
| **MDAT Subdomain Scores^d^** | | | | | | | | | | | | | |
| MDAT Gross Motor | Antenatal | 83/138 (60.1) | 15.6 (1.5) | 16.1 (1.7) | 0.315 | 205/367 (55.9) | 20.3 (1.4) | 20.1 (1.7) | 0.199 | 189/338 (55.9) | 21.4 (1.4) | 21.4 (1.3) | 0.616 |
|  | *14–23* | 57/124 (46.0) | 16.0 (1.5) | 15.9 (1.6) | 0.876 | 127/303 (41.9) | 20.2 (1.4) | 20.1 (1.8) | 0.676 | 112/274 (40.9) | 21.4 (1.3) | 21.4 (1.4) | 0.594 |
|  | *>23–28* | 26/124 (21.0) | 15.9 (1.5) | 16.3 (2.0) | 0.321 | 62/331 (18.7) | 20.2 (1.5) | 20.1 (1.9) | 0.463 | 59/310 (19.0) | 21.4 (1.3) | 21.4 (1.3) | 0.797 |
|  | *>28–33* | 25/137 (18.2) | 15.9 (1.6) | 16.3 (1.5) | 0.326 | 67/358 (18.7) | 20.2 (1.5) | 20.2 (1.9) | 0.771 | 65/331 (19.6) | 21.4 (1.3) | 21.4 (1.5) | 0.842 |
|  | *> 33-37* | 17/117 (14.5) | 16.0 (1.5) | 15.6 (1.7) | 0.269 | 56/305 (18.4) | 20.3 (1.4) | 19.9 (2.0) | 0.102 | 53/276 (19.2) | 21.5 (1.3) | 21.3 (1.4) | 0.279 |
|  | Placental | 34/128 (26.6) | 16.1 (1.7) | 15.9 (1.5) | 0.613 | 98/330 (29.7) | 20.3 (1.4) | 20.2 (2.0) | 0.613 | 91/307 (29.6) | 21.5 (1.3) | 21.2 (1.4) | 0.041 |
|  |  |  |  |  |  |  |  |  |  |  |  |  |  |
| MDAT Fine Motor | Antenatal | 83/138 (60.1) | 16.4 (1.3) | 16.5 (1.8) | 0.670 | 205/367 (55.9) | 19.2 (1.4) | 19.4 (1.5) | 0.175 | 189/338 (55.9) | 23.3 (1.9) | 23.5 (2.0) | 0.265 |
|  | *14–23* | 57/124 (46.0) | 16.4 (1.4) | 16.5 (1.8) | 0.641 | 127/303 (41.9) | 19.2 (1.3) | 19.5 (1.5) | 0.077 | 112/274 (40.9) | 23.4 (1.9) | 23.6 (2.0) | 0.322 |
|  | *>23–28* | 26/124 (21.0) | 16.4 (1.7) | 16.9 (1.4) | 0.114 | 62/331 (18.7) | 19.2 (1.3) | 19.4 (1.5) | 0.449 | 59/310 (19.0) | 23.4 (1.9) | 23.5 (2.1) | 0.697 |
|  | *>28–33* | 25/137 (18.2) | 16.4 (1.4) | 16.7 (2.4) | 0.395 | 67/358 (18.7) | 19.3 (1.3) | 19.3 (1.4) | 0.989 | 65/331 (19.6) | 23.4 (1.9) | 23.4 (2.0) | 0.970 |
|  | *> 33-37* | 17/117 (14.5) | 16.5 (1.7) | 16.4 (1.1) | 0.718 | 56/305 (18.4) | 19.3 (1.3) | 19.3 (1.7) | 0.884 | 53/276 (19.2) | 23.5 (1.8) | 23.3 (2.0) | 0.415 |
|  | Placental | 34/128 (26.6) | 16.5 (1.7) | 16.3 (1.4) | 0.547 | 98/330 (29.7) | 19.2 (1.3) | 19.4 (1.6) | 0.362 | 91/307 (29.6) | 23.4 (1.9) | 23.5 (2.1) | 0.813 |
|  |  |  |  |  |  |  |  |  |  |  |  |  |  |
| MDAT Language | Antenatal | 83/138 (60.1) | 10.5 (1.6) | 10.4 (2.8) | 0.856 | 205/367 (55.9) | 12.7 (2.1) | 12.8 (1.9) | 0.611 | 189/338 (55.9) | 18.8 (3.2) | 18.7 (3.0) | 0.782 |
|  | *14–23* | 57/124 (46.0) | 10.5 (1.8) | 10.4 (3.1) | 0.812 | 127/303 (41.9) | 12.8 (2.1) | 12.7 (1.8) | 0.431 | 112/274 (40.9) | 18.9 (3.1) | 18.8 (2.9) | 0.852 |
|  | *>23–28* | 26/124 (21.0) | 10.3 (1.9) | 10.6 (2.2) | 0.466 | 62/331 (18.7) | 12.7 (1.9) | 13.1 (2.0) | 0.136 | 59/310 (19.0) | 18.8 (3.0) | 18.8 (3.6) | 0.982 |
|  | *>28–33* | 25/137 (18.2) | 10.5 (2.4) | 10.2 (2.2) | 0.660 | 67/358 (18.7) | 12.6 (2.0) | 13.2 (2.0) | 0.050 | 65/331 (19.6) | 18.6 (3.1) | 19.4 (3.1) | 0.073 |
|  | *> 33-37* | 17/117 (14.5) | 10.4 (1.7) | 10.3 (4.9) | 0.845 | 56/305 (18.4) | 12.8 (2.0) | 12.5 (2.0) | 0.318 | 53/276 (19.2) | 19.0 (3.0) | 18.3 (3.0) | 0.143 |
|  | Placental | 34/128 (26.6) | 10.6 (2.7) | 9.7 (1.1) | 0.054 | 98/330 (29.7) | 12.7 (2.0) | 12.9 (1.9) | 0.460 | 91/307 (29.6) | 18.9 (3.0) | 18.4 (3.5) | 0.225 |
|  |  |  |  |  |  |  |  |  |  |  |  |  |  |
| MDAT Social | Antenatal | 83/138 (60.1) | 16.1 (1.7) | 15.6 (1.9) | 0.133 | 205/367 (55.9) | 18.5 (2.4) | 18.9 (2.6) | 0.114 | 189/338 (55.9) | 25.9 (2.8) | 26.0 (2.8) | 0.881 |
|  | *14–23* | 57/124 (46.0) | 16.1 (1.8) | 15.5 (1.9) | 0.123 | 127/303 (41.9) | 18.4 (2.3) | 19.1 (2.8) | 0.018 | 112/274 (40.9) | 26.2 (2.6) | 26.0 (2.9) | 0.455 |
|  | *>23–28* | 26/124 (21.0) | 15.8 (1.7) | 15.8 (1.9) | 0.926 | 62/331 (18.7) | 18.7 (2.5) | 19.2 (2.7) | 0.124 | 59/310 (19.0) | 26.0 (2.8) | 25.7 (3.1) | 0.400 |
|  | *>28–33* | 25/137 (18.2) | 15.9 (1.8) | 15.3 (1.9) | 0.119 | 67/358 (18.7) | 18.7 (2.5) | 18.9 (2.4) | 0.534 | 65/331 (19.6) | 25.8 (2.9) | 26.5 (2.5) | 0.110 |
|  | *> 33-37* | 17/117 (14.5) | 15.8 (1.8) | 16.2 (1.7) | 0.394 | 56/305 (18.4) | 18.8 (2.5) | 18.6 (2.6) | 0.637 | 53/276 (19.2) | 26.1 (2.8) | 25.9 (2.3) | 0.595 |
|  | Placental | 34/128 (26.6) | 15.9 (1.8) | 15.4 (1.9) | 0.161 | 98/330 (29.7) | 18.7 (2.5) | 18.9 (2.5) | 0.443 | 91/307 (29.6) | 26.0 (2.8) | 25.8 (2.7) | 0.471 |
| ^a^Malaria exposures defined as antenatal malaria (peripheral PCR-confirmed malaria at any point during pregnancy) or placental malaria (positive placental histology or placental PCR at delivery). The former is stratified by gestational age (*weeks*) at time of PCR-confirmed infection.  ^b^n/N(%): malaria positive women as a percentage of total women (N) with existing data for both the respective neurocognitive score and malaria variable. ^c^p-values represent unadjusted ordinary least squares regression before correction for multiple comparisons. p-values that remain significant after Holm-Bonferonni adjustment for multiple comparisons across age at neurocognitive assessment and malaria exposure variables (n=18 for MDAT; n=12 for MCAB-CDI) are marked by an asterisk. ^d^Scores expressed as mean (standard deviation). ^e^Age of MCAB-CDI first assessment was 18 months. Abbreviations: Malawi Development Assessment Tool (MDAT), McArthur Bates Communication Development Inventory (MCAB-CDI). | | | | | | | | | | | | | |
