# Supplementary material for: Neurocognitive outcomes in Malawian children exposed to malaria during pregnancy: An observational birth cohort study
Source: PLoS Med. 2021 Sep 28;18(9):e1003701. doi: 10.1371/journal.pmed.1003701 (PMC8478258; doi:10.1371/journal.pmed.1003701)
Supplement: S7 Table — (DOCX) [file pmed.1003701.s010.docx]

| **Supplementary Table 7. Descriptive characteristics of PAMaNeD study cohort versus parent trial cohort** | | | | | | | |
| --- | --- | --- | --- | --- | --- | --- | --- |
|  | | | | | **Parent Cohort** | **PAMaNeD** |  |
| n | | | | | 1452 | 421 |  |
| **Baseline Characteristics** | | | | | **n (%)^a^ or median [IQR]** | | **p-value^b^** |
| Maternal age (years) | | | | | 21 [18, 26] | 21 [19, 25] | 0.534 |
| Gestational age at enrolment (weeks) | | | |  | 21.0 [19.0, 23.0] | 19.7 [17.9, 22.1] | <0.001 |
| Socioeconomic status (tertile) | | | | 1 | 498 (34.3) | 125 (29.8) | 0.208 |
|  | | | | 2 | 482 (33.2) | 145 (34.6) |  |
|  | | | | 3 | 470 (32.4) | 149 (35.6) |  |
| Primigravidity | |  | | | 491 (33.8) | 144 (34.2) | 0.935 |
| Maternal education status (tertile) | 1 | | | | 449 (31.0) | 125 (29.8) | 0.743 |
|  | 2 | | | | 762 (52.6) | 229 (54.7) |  |
|  | 3 | | | | 239 (16.5) | 65 (15.5) |  |
| **Perinatal Characteristics** | | | | | | | |
| Gestational age at delivery (weeks) | | | | | 38.3 [37.0, 39.6] | 38.7 [37.4, 39.9] | <0.001 |
| Birth weight (g) | | | | | 2954 [2600, 3200] | 3000 [2700, 3200] | 0.010 |
| Sex | | | Male | | 666 (50.7) | 211 (50.1) | 0.709 |
|  | | | Female | | 647 (49.3) | 210 (49.9) |  |
| Preterm birth (< 37 weeks gestation) | | | | | 278 (21.7) | 71 (16.9) | 0.040 |
| Low birth weight (< 2500 g) | | | | | 146 (11.9) | 28 (6.8) | 0.006 |
| Small-for-gestational age | | | | | 142 (11.6) | 33 (8.1) | 0.059 |
| **Malaria Status** | | | | | | | |
| Antenatal malaria positive | | | | | 893 (68.0) | 240 (57.3) | <0.001 |
| Placental malaria positive | | | | | 450 (37.6) | 112 (29.6) | 0.006 |
| Cord PCR positive at delivery | | | | | 156 (13.8) | 35 (9.8) | 0.064 |
| ^a^ n(%) expressed as percent of women with existing data for respective variable. ^b^p-value of Chi-square, or Wilcoxon rank-sum test. Abbreviations: Interquartile Range (IQR), The Effect of Pregnancy Associated Malaria on Early Childhood Neurocognitive Development: an Observational Birth Cohort Study (PAMaNeD). | | | | | | | |
