# Supplementary material for: Neurocognitive outcomes in Malawian children exposed to malaria during pregnancy: An observational birth cohort study
Source: PLoS Med. 2021 Sep 28;18(9):e1003701. doi: 10.1371/journal.pmed.1003701 (PMC8478258; doi:10.1371/journal.pmed.1003701)
Supplement: S1 Text — (DOCX) [file pmed.1003701.s011.docx]

**Supplementary Text 1: Supplementary Methods**

***Further details of testing personnel reliability checks***

Every three months, reliability of testing personnel was assessed on a sub-selection of ten participants. In total, 78 participants were re-tested by the gold standard trainer and one of six randomly chosen testing personnel. Testing personnel achieved a mean score of 97.8% reliability.

***Further details of correction for multiple comparisons***

P-values were adjusted for multiple comparisons using the Holm-Bonferonni method. In the bivariate analysis of neurocognitive outcomes (at 12, 18, and 24 months), p-values were adjusted for the number of testing age comparisons multiplied by the number of malaria comparisons (e.g. 3 testing ages x 6 malaria variables; n=18). In the longitudinal analysis of neurocognitive outcomes, p-values were adjusted for the number of malaria comparisons (n=6), or the number of inflammatory analytes multiplied by the number of inflammatory analyte timing comparisons (3 analytes x 3 gestational windows; n=9).

***Further details of neurocognitive testing methods (A-not-B and Delayed Inhibition methods)***

*A-not-B test.* At 18 and 24 months, the A-not-B task was used to assess working memory and object permanence. For this task, an object is hidden in one of two locations (A or B), as the child watches. After a delay, the child must indicate the object’s location. This task was repeated for 10 trials, and the task was scored as the proportion of correct trials. Across trials, the object’s location was switched according to a strict protocol to check for perseveration error. We modelled A-not-B test proportional data with weighted quasibinomial regression. Quasibinomial regression was used to account for overdispersion of data. All scores were included in the analysis, but regression models of A-not-B data were weighted for the number of completed trials.

*Delayed Inhibition.* At 18 and 24 months, the delayed inhibition task was used to test self-control and impulsivity. During this task, the child is presented with a treat (either a biscuit or a sweet) and must wait 2 minutes before taking it. Delayed inhibition is scored as a binary outcome for each treat: successfully waited 2 minutes (complete delay) or did not wait 2 minutes (incomplete delay). The relative risk of failing the delayed inhibition task was calculated using log-binomial regression (or log-Poisson regression in cases where the model did not converge).
